# m^6^A Reader hnRNPA2B1 Modulates Late Pachytene Progression in Male Meiosis Through Post‐Transcriptional Control

**DOI:** 10.1002/advs.202506600

**Published:** 2025-07-28

**Authors:** Lisha Yin, Yuting Zhang, Bingqian Zhang, Jin Zhang, Mengneng Xiong, Nan Jiang, Jinxin Xiao, Huihui Gao, Wenjing Xiong, Xiaoli Wang, Fengli Wang, Shuiqiao Yuan

**Affiliations:** ^1^ Institute of Reproductive Health Tongji Medical College Huazhong University of Science and Technology Wuhan 430030 China; ^2^ Reproductive Medicine Center Renmin Hospital of Wuhan University Wuhan 430030 China; ^3^ Department of Obstetrics and Gynecology The Central Hospital of Wuhan Tongji Medical College Huazhong University of Science and Technology Wuhan 430014 China; ^4^ Laboratory of Animal Center Huazhong University of Science and Technology Wuhan 430030 China; ^5^ Shenzhen Huazhong University of Science and Technology Research Institute Shenzhen 518057 China

**Keywords:** meiosis, m^6^A, hnRNPA2B1, pachytene spermatocyte, post‐transcription regulation, spermatogenesis

## Abstract

N^6^‐methyladenosine (m^6^A) reader proteins have been demonstrated to be involved in numerous biological processes. However, the regulatory mechanism of specific m^6^A reader proteins during mammalian meiotic processes remains largely elusive. Here, this study identified hnRNPA2B1 as an m^6^A reader protein that plays a critical role in meiotic pachytene progression using a tamoxifen‐induced knockout mouse model. Deletion of hnRNPA2B1 in spermatocytes disrupts homologous recombination and synapsis, with the mislocalization of double‐strand break (DSB) repair proteins beyond the chromosome axes in pachytene spermatocytes. Multi‐omics analyses revealed extensive dysregulation of the transcriptome and proteome in hnRNPA2B1‐deficient spermatocytes, particularly affecting genes involved in chromosome organization, meiotic cell cycle, and DNA damage response, thereby triggering the pachytene checkpoint for cell elimination. In vitro luciferase assays confirmed that hnRNPA2B1 directly targets several meiosis‐related transcripts (e.g., *Ep400*, *Rrs1*, etc.) in an m^6^A‐dependent manner to regulate their expression. Furthermore, this finding demonstrates that hnRNPA2B1 biologically interacts with mRNA processing regulators and translation factors (e.g., eIF4G3, RPS3, RPL13, DDX5, YTHDC2) and functions as a post‐transcriptional factor essential for pachytene progression during male meiosis. Collectively, this study underscores the critical role of the m^6^A reader hnRNPA2B1 in the pachytene checkpoint and advances our understanding of the regulatory mechanisms underlying male meiosis.

## Graphical abstract

As an m^6^A reader, hnRNPA2B1 predominantly regulates specific transcripts (e.g., *Ep400*, *Rrs1*) that are essential for chromosome organization and remodeling in an m^6^A‐dependent manner. Furthermore, it is demonstrated to interact with factors involved in mRNA processing and translation. This dual functionality is pivotal for ensuring the proper occurrence of chromosomal events and supporting late pachytene progression during male meiosis.

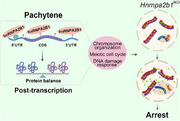

## 1 Introduction

Meiosis is a fundamental biological process that enables genetic diversity through the exchange of parental genetic material. This process involves multiple chromosomal events, including DNA strand breakage (DSB) formation, homologous recombination, synapsis, crossover, desynapsis, and chromosome alignment and segregation. Meiotic checkpoints ensure that only cells with coordinated chromosomal activity and successful recombination proceed to produce haploid spermatids. The pachytene checkpoint, also known as the meiotic recombination checkpoint, eliminates spermatocytes with defective recombination and synapsis. While the components of this checkpoint are well‐characterized in yeast, including DNA damage checkpoint proteins (*Dcd1*, *Chk1*, *Mec1/3*), meiotic chromosome proteins (*Hop1/2*, *Mek1*, *Red1*, *Zip1*), recombination proteins (*Dmc1*, *Mlh1*, *Msh5*, *Rad51*), chromatin‐silencing factors (*Sir2*, *Pch2*, *Dot1*), and cell cycle proteins (*Cdc28*, *Clb1*, *Ndt80*),^[^ ^1^ ^]^ the precise molecular mechanisms remain to be fully elucidated in mice.

The role of *N6*‐methyladenosine (m^6^A), the most abundant RNA modification involved in mRNA splicing, stability, degradation, and translation, has recently gained attention in spermatogenesis research. m^6^A modification is regulated by “writers” (e.g., METTL3/14/16, WTAP, KIAA1429), “erasers” (e.g., FTO, ALKHB5), and “readers” (e.g., YTH domain proteins, hnRNPs, IGF2BP family members, FMR1, PRRC2A).^[^ ^2^ ^]^ The importance of m^6^A‐related proteins in spermatogenesis has been demonstrated in numerous studies. For example, *Vasa*‐Cre‐induced deletion of *Mettl3/14* leads to SSC depletion, while *Stra8*‐Cre‐induced double deletion disrupts spermiogenesis, due to dysregulated translation of transcripts essential for SSC proliferation/differentiation or spermiogenesis.^[^ ^3^ ^]^ Similarly, our recent studies have shown that loss of *Mettl16* in spermatogonia or spermatocytes leads to germ cell apoptosis and male infertility.^[^ ^4^, ^5^ ^]^ FTO deletion impairs spermatogonial proliferation, germ cell survival, and Leydig cell maturation.^[^ ^6^ ^]^ While *Ythdc2* global knockout mice are arrested at the zygotene stage due to alterations in translation efficiency (TE) and mRNA abundance,^[^ ^7^ ^]^ *Ddx4*‐Cre^ERT2^ inducible *Ythdc2* knockout mice show late pachytene spermatocyte loss caused by an altered transcriptome.^[^ ^8^ ^]^ PRRC2A‐null spermatocytes show defects in meiotic sex chromosome inactivation (MSCI) and metaphase spindle organization.^[^ ^9^ ^]^ The phenotypes in these genetically modified mice are associated to varying degrees with the m^6^A modification, highlighting the critical role of the m^6^A modification in spermatogenesis.

Of note, many members of the hnRNPs have also been implicated in spermatogenesis. Our previous studies have shown that *Stra8/Amh*‐Cre‐induced hnRNPH1 deletion results in spermatogenesis arrest and splicing defects,^[^ ^10^, ^11^ ^]^ while hnRNPU has been shown to regulate splicing and transcription factors *Sox8/9* to control spermatogenesis process.^[^ ^12^, ^13^ ^]^ Furthermore, we and other researchers have demonstrated that the deficiencies in hnRNPC or hnRNPK in germ cells/Sertoli cells could compromise the blood‐testis barrier and meiotic progression.^[^ ^14^, ^15^, ^16^ ^]^ While our earlier research established that hnRNPA2B1 can repress the disassembly of arsenite‐induced stress granules and plays an essential role in male fertility,^[^ ^17^ ^]^ its role in male meiosis remains to be elucidated.

Here, we identified a novel function of the m^6^A reader hnRNPA2B1 in male meiosis. Utilizing a *Ddx4*‐Cre^ERT2^ inducible knockout mouse model, we have demonstrated that hnRNPA2B1 modulates the expression of specific transcripts that are essential for chromosome organization (e.g., *Ep400*, *Rrs1*) in an m^6^A‐dependent manner, and interacts with several factors essential for mRNA processing and translation (e.g., eIF4G3, RPS3, RPL13, DDX5, YTHDC2), to regulate pachytene progression. These findings provide critical insights into the regulatory network of m^6^A reader protein governing late meiosis progression and completion, advancing our understanding of male meiosis.

## 2 Results

### 2.1 Conditional Deletion of hnRNPA2B1 Results in Meiotic Arrest at Pachytene Stage

**Figure 1:**
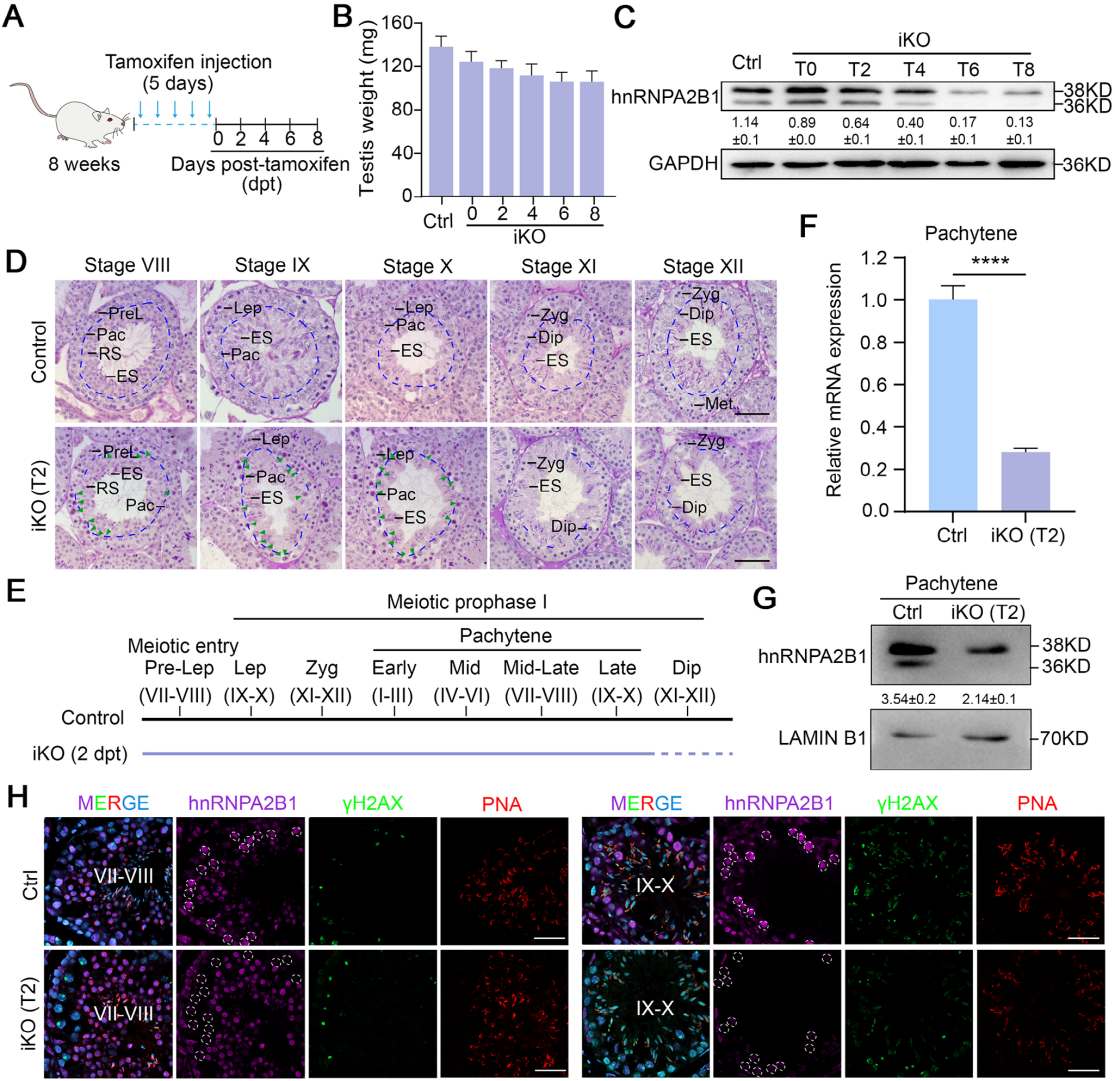
hnRNPA2B1 is essential for late meiosis progression. (A) Schematics of *Hnrnpa2b1^flox/flox^Ddx4‐Cre* ^ERT2^ inducible knockout mouse generation are shown. Control (*Hnrnpa2b1* ^*flox/flox*^) and *Hnrnpa2b1^flox/flox^Ddx4‐Cre* ^ERT2^ (*Hnrnpa2b1* ^iKO^ or iKO) adult mice were treated with five consecutive days tamoxifen injection, and the testis tissues were collected at 0 (T0), 2, 4, 6, and 8 days post‐injection. (B) Histogram showing testis weight in Control (Ctrl) and iKO mice at various time points subsequent to tamoxifen injection. *n* = 4 mice. (C) Western blot assays and quantification of hnRNPA2B1 in Ctrl and iKO testes at T0, T2, T4, T6, and T8. GAPDH serves as loading control. Quantifications of hnRNPA2B1 account for both two bands. The quantified data are presented as mean ± SD. *n* = 3 mice. (D) Histological analysis of seminiferous tubules at stage VIII‐XII in Ctrl and iKO mice at T2. The blue lines meant the boundary of pachytene/diplotene and spermatids. The green arrowheads meant the dead cells. Abbreviations: PreL, preleptotene spermatocytes; Lep, leptotene spermatocytes; Zyg, zygotene spermatocytes; Pac, pachytene spermatocytes; Dip, diplotene spermatocytes; Met, metaphase cells; RS, round spermatids; ES, elongating/elongated spermatids. Scale bars = 50 µm. (E) Schematics of meiosis progression in Ctrl and iKO mice at T2. Dashed lines indicated obvious cell loss. (F) RT‐qPCR analysis to confirm the knockdown of *Hnrnpa2b1* in pachytene spermatocytes isolated from Ctrl and *Hnrnpa2b1* ^iKO^ mice at T2. *n* = 3 mice. The quantified data were presented as mean ± SD. ****p < 0.0001. (G) Western blot assay and quantification to confirm the knockdown of hnRNPA2B1 in pachytene spermatocytes isolated from Ctrl and iKO mice at T2. Quantifications of hnRNPA2B1 account for both two bands. The quantified data are presented as mean ± SD. *n* = 4 mice. (H) Immunofluorescence to confirm the obvious loss of hnRNPA2B1 in pachytene spermatocytes of iKO mice at T2. Representative images of tubules at Stage VII‐VIII and Stage IX‐X were shown. γH2AX was used for spermatocytes identification and PNA was used to identify the stage of the seminiferous tubules. White lines indicate pachytene spermatocytes. Scale bars = 50 µm.

In the preceding study, hnRNPA2B1 was identified as being expressed in various types of spermatogenic cells, including Sertoli cells, spermatogonia, spermatocytes, and round spermatids.^[^ ^17^ ^]^ Furthermore, it was found that *Hnrnpa2b1* global knockout (gKO) males exhibited a Sertoli‐cell‐only phenotype from postnatal day 14 (P14) onward.^[^ ^17^ ^]^ In the present study, hnRNPA2B1 was found to exist in both the nucleus and cytoplasm of germ cells, and be highly enriched in pachytene and diplotene spermatocytes in both humans and mice (Figure S1A–C, Supporting Information), suggesting that hnRNPA2B1 may function in meiotic prophase I progression. To circumvent the early meiotic arrest that has been observed in global knockout *Hnrnpa2b1* mutants, a tamoxifen‐induced knockout mouse model driven by *Ddx4*‐Cre^ERT2^ was created, thereby avoiding both pre‐meiotic and early meiotic arrest (**Figure** **1**A). Intraperitoneal tamoxifen injection into *Hnrnpa2b1^flox/flox^Ddx4*‐Cre^ERT2^ (referred to as *Hnrnpa2b1* ^iKO^ or iKO) and *Hnrnpa2b1^flox/flox^* adult mice (referred to as Ctrl) was performed for five consecutive days, and testes at various time points post‐tamoxifen treatment (T0, T2, T4, T6, or T8) were harvested for analysis (Figure 1A). The results obtained revealed a gradual decrease in testis weight (Figure 1B), concomitant with a decline in the abundance of hnRNPA2B1 protein (both A2 and B1 isoforms) in testis lysate, in *Hnrnpa2b1^iKO^* mice (Figure 1C). These findings suggest that the tamoxifen‐induced germ cell‐specific knockout mouse model has been successfully generated.

In order to explore the function of hnRNPA2B1 in meiosis, we conducted histological analyses using testes from control and *Hnrnpa2b1^iKO^* adult male mice. PAS staining revealed that, at T0, no obvious defects were observed in *Hnrnpa2b1^iKO^* mice (Figure S1D,E, Supporting Information). However, at T2, numerous apoptotic pachytene spermatocytes, characterized by their larger size and deeper staining,^[^ ^18^ ^]^ were present in the seminiferous tubules of *Hnrnpa2b1^iKO^* mice (Figure 1D). Of particular note was the accumulation of these apoptotic pachytene cells at stages VIII, IX, and X (late pachytene spermatocytes) and a significant reduction in the number of diplotene spermatocytes was observed in *Hnrnpa2b1^iKO^* mice at T2 (Figure 1D,E), suggesting defects in late meiosis in *Hnrnpa2b1^iKO^* mice. Furthermore, at T4, T6, and T8, the number of late pachytene cells in seminiferous tubules at stages VIII‐XII was dramatically reduced, and the diplotene cells at stage XI‐XII were nearly absent (Figure S1D,E, Supporting Information), suggesting a progressive loss of spermatocytes in *Hnrnpa2b1^iKO^* mice following tamoxifen treatment. Concurrently, round/elongated spermatids from the preceding wave of spermatogenesis persisted in *Hnrnpa2b1^iKO^* mice, thereby further substantiating a meiotic blockade caused by hnRNPA2B1 loss in the present wave. Due to the scarcity of late meiotic cells in the seminiferous tubules of *Hnrnpa2b1^iKO^* mice at T4 and subsequent time points (Figure S1D,E, Supporting Information), and the low knockout efficiency at T0 (Figure S1F, Supporting Information), T2 was chosen as the optimal time point for further investigation. At T2, both the mRNA and protein levels of hnRNPA2B1 were reduced in *Hnrnpa2b1^iKO^* pachytene spermatocytes (Figure 1F,G). Further immunofluorescence (IF) assay also confirmed a significant loss of hnRNPA2B1 in *Hnrnpa2b1^iKO^* late pachytene spermatocytes at T2–T6 (Figure 1H; Figure S1F, Supporting Information). Collectively, these observations indicate that hnRNPA2B1 is indispensable for male meiosis, particularly during pachytene progression.

### 2.2 Elimination of hnRNPA2B1‐Deficient Spermatocytes at the Pachytene Stage

**Figure 2:**
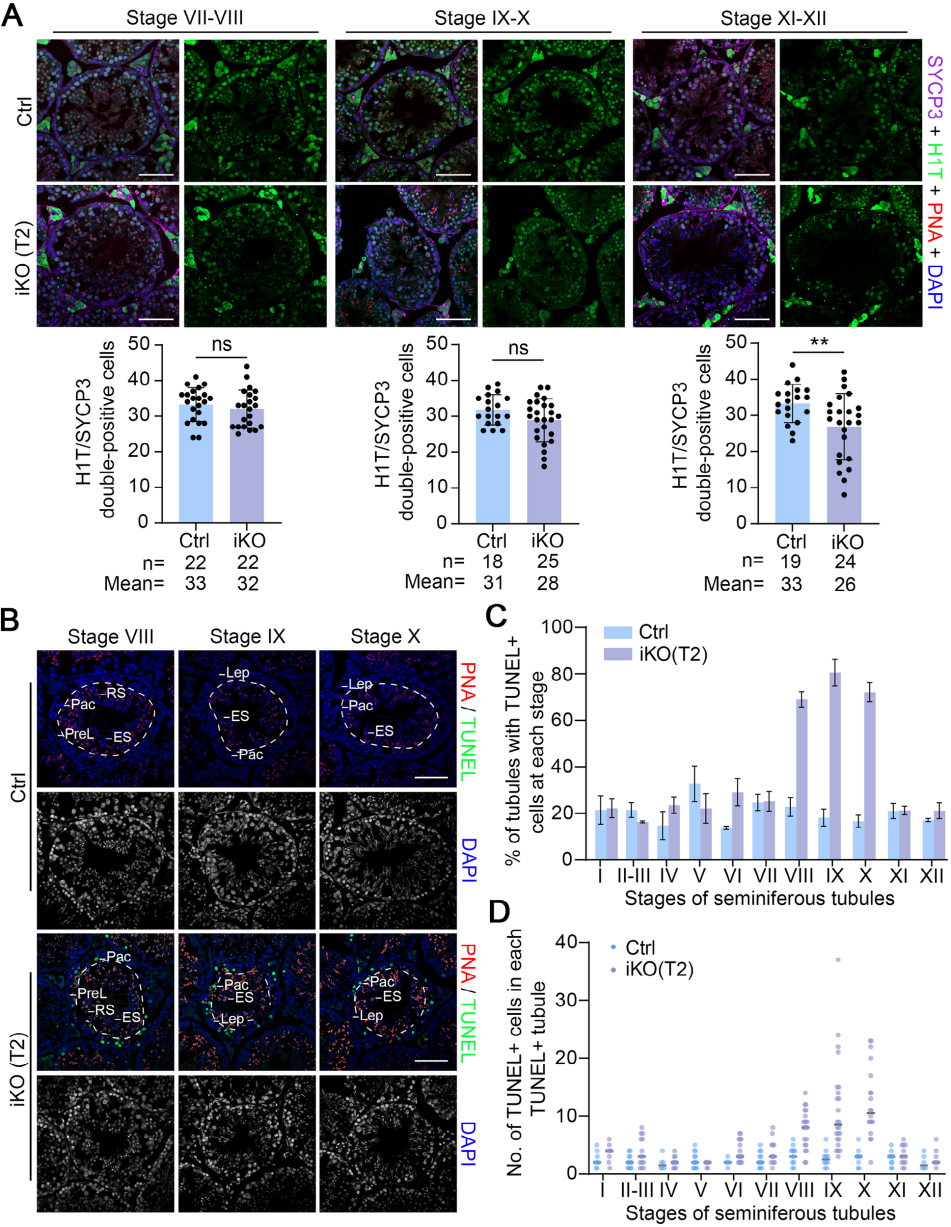
hnRNPA2B1‐deficient spermatocytes undergo apoptosis at the late pachytene stage. (A) Representative immunofluorescence images and quantification of SYCP3/H1T for stage VII‐VIII, stage IX‐X, and stage XI‐XII seminiferous tubules in Ctrl and iKO mice at T2 are shown. PNA was used to identify the stage of the seminiferous tubules. n, the total number of seminiferous tubules from Ctrl and *Hnrnpa2b1* ^iKO^ testes. Mean, the average number of H1T/SYCP3 double‐positive cells in seminiferous tubules. Three males per genotype (Ctrl and iKO) were analyzed. Scale bars = 50 µm. The quantified data are presented as mean ± SD. **p < 0.01; ns, not significant. (B) TUNEL analysis of testis sections from Ctrl and iKO mice at T2. The dashed lines delineate the boundary of pachytene cells (the outer layer) and spermatids (the inner layer). PNA was used to identify the stage of the seminiferous tubules. Abbreviations: PreL, preleptotene; Lep, leptotene; Pac, pachytene; RS, round spermatids; ES, elongating spermatids. Scale bars = 50 µm. (C) The percentage of TUNEL‐positive tubules from Ctrl and iKO testes at T2 is shown. Two males per genotype (Ctrl and iKO) were analyzed. More than 200 tubules were counted for each mouse. (D) Quantification of TUNEL‐positive cells in TUNEL‐positive tubules from Ctrl and iKO testes at T2. Two males per genotype (Ctrl and iKO) were analyzed. More than 200 tubules were counted for each mouse.

To determine the precise extent of spermatocyte loss, an immunostaining assay was performed using an H1T antibody, a specific marker for mid‐late pachytene cells, diplotene cells, and round spermatids, followed by stage‐specific quantification of H1T^+^/SYCP3^+^ cells. The results showed that in control mice, the average number of late pachytene cells was ≈33 at stage VII‐VIII and ≈31 at stage IX‐X, while diplotene cells numbered ≈33 at stage XI‐XII. In contrast, *Hnrnpa2b1* ^iKO^ mice showed a significant reduction in the diplotene cell population at T2 for stage XI‐XII, with the average number dropping to ≈26 per tubule (**Figure** **2**A). This loss of spermatocytes was severely progressive in *Hnrnpa2b1* ^iKO^ mice at later time points, with cell numbers further reduced to ≈10 at T4 and ≈2 at T6 for stage VII‐VIII, ≈7 at T4 and ≈1 at T6 for stage IX‐X, ≈6 at T4 and ≈1 at T6 for stage XI‐XII (Figure S2A, Supporting Information), demonstrating a pronounced and progressive loss of late pachytene and diplotene spermatocytes in *Hnrnpa2b1* ^iKO^ mice.

The significant depletion of late pachytene and diplotene spermatocytes in seminiferous tubules during stages VIII‐XII in *Hnrnpa2b1^iKO^* mice suggests that spermatocytes perish at a particular stage. To ascertain the precise timing of cell death in *Hnrnpa2b1^iKO^* mice, a TUNEL assay was conducted in conjunction with PNA staining (Figure 2B). The results obtained demonstrated that tubules exhibiting with TUNEL‐positive spermatocytes were predominantly present at stages VIII‐X (Figure 2C; Figure S2B,C, Supporting Information), indicating late pachytene apoptosis. Furthermore, it was observed that the average number of TUNEL‐positive spermatocytes in both the Ctrl and iKO mice at T2 was significantly elevated at stages VIII‐X (Stage VIII: ≈7 in iKO vs ≈3 in Ctrl; Stage IX: ≈11 in iKO vs ≈2 in Ctrl; Stage X: ≈12 in iKO vs ≈2 in Ctrl) (Figure 2D; Figure S2D, Supporting Information). Consequently, these data suggest that the loss of late pachytene and diplotene spermatocytes in *Hnrnpa2b1* ^iKO^ mice was due to apoptosis.

### 2.3 hnRNPA2B1‐Depleted Pachytene Spermatocytes Displayed Abnormal Synapsis, Recombination, and Crossover

**Figure 3:**
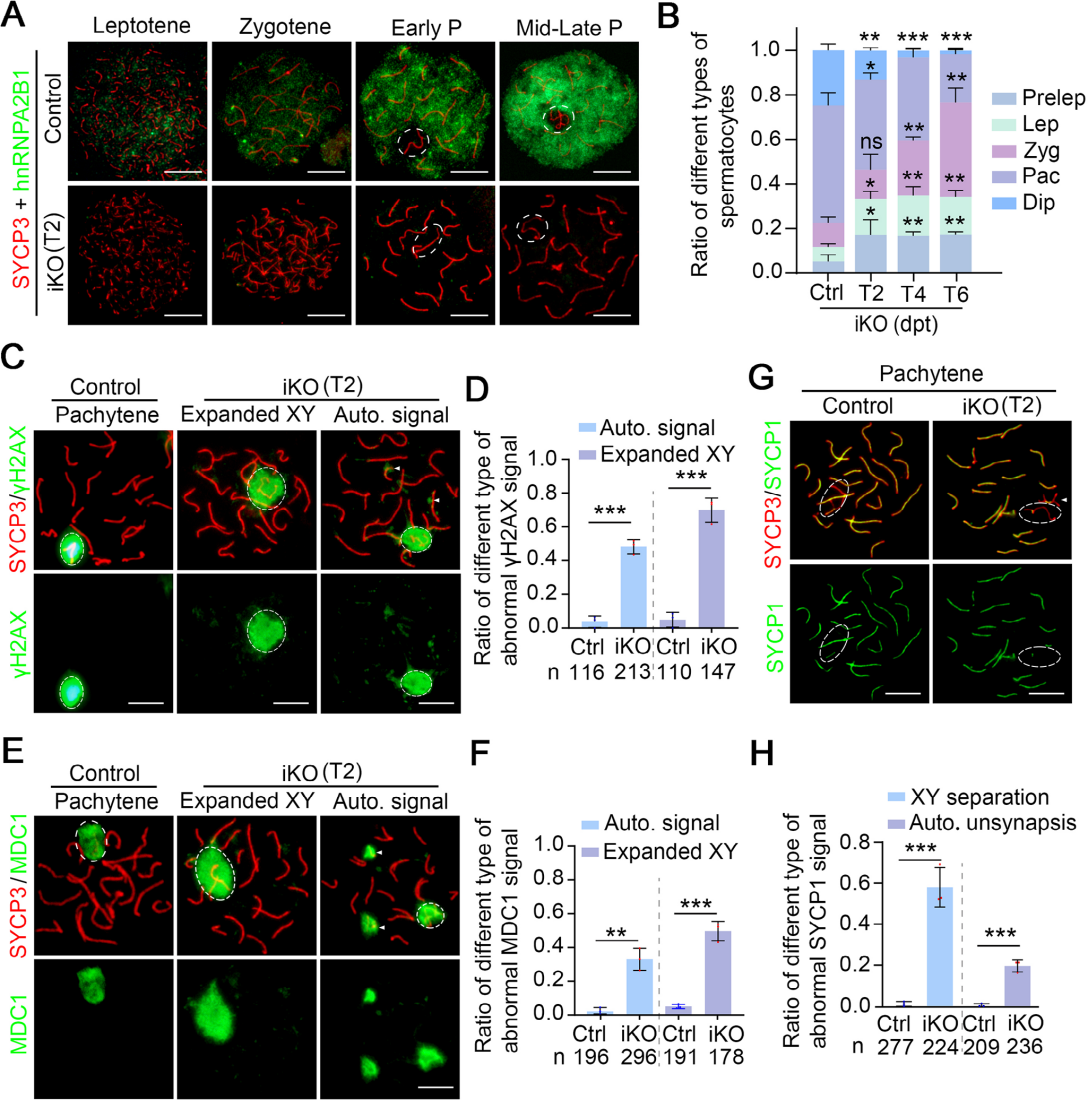
Ablation of hnRNPA2B1 in spermatocytes impairs meiotic progression. (A) Representative images of chromosome spread assay showing the efficiency of the hnRNPA2B1 knockout in different substages of meiotic prophase I in iKO mice at T2. The white circles indicate XY bodies in early pachytene (Early P) and mid‐late pachytene (Mid‐Late P). Scale bars = 10 µm. (B) The ratio of different type of spermatocytes in Ctrl and iKO mice at T2, T4, and T6. The quantified data are presented as mean ± SD. *p < 0.05, **p < 0.01, ***p < 0.001. ns, not significant. *n* = 3 mice. (C,D) Representative images (C) and quantification (D) of chromosome spread analysis for DSB repair marker γH2AX in pachytene spermatocytes in Ctrl and iKO mice at T2 are shown. The white circles indicate XY bodies. Scale bars = 10 µm. The γH2AX signal not restricted to region surrounding sex chromosomes but instead spreading to adjacent autosomes was defined as “expanded sex body.” Auto. signal refers to pachytene spermatocytes exhibiting autosomal γH2AX signal. Arrowheads indicate the autosomes with γH2AX signal. Histogram in (D) showing the ratio of different type of abnormal γH2AX signal. n, the total number of quantified pachytene spermatocytes. The quantified data are presented as mean ± SD. ***p < 0.001. Three males per genotype (Ctrl and iKO) were analyzed. (E,F) Representative images (E) and quantification (F) of chromosome spread analysis for MDC1 in pachytene spermatocytes in Ctrl and iKO mice at T2 are shown. The white circles indicate XY bodies. Scale bars = 10 µm. The MDC1 signal not restricted to region surrounding sex chromosomes but instead spreading to adjacent autosomes was defined as “expanded sex body.” Auto. signal refers to pachytene spermatocytes exhibiting autosomal γH2AX signal. Arrowheads indicate autosomes with MDC1 signal. Histogram in (F) showing the ratio of different type of abnormal MDC1 signal. n, the total number of quantified pachytene spermatocytes. The quantified data are presented as mean ± SD. **p < 0.01, ***p < 0.001. Three males per genotype (Ctrl and iKO) were analyzed. (G‐H) Representative images (G) and quantification (H) of chromosome spread analysis for synapsis marker SYCP1 in pachytene spermatocytes in Ctrl and iKO mice at T2 are shown. The white circles indicate XY bodies. Arrowheads mean autosomes with incomplete SYCP1 localization. Scale bars = 10 µm. Histogram in (H) showing the ratio of different type of abnormal SYCP1 signal. Auto. unsynapsis refers to pachytene spermatocytes exhibiting incomplete SYCP1 localization across autosomes. n, the total number of quantified pachytene spermatocytes. The quantified data are presented as mean ± SD. ***p < 0.001. Three males per genotype (Ctrl and iKO) were analyzed.

To investigate the specific defects present in pachytene spermatocytes in *Hnrnpa2b1^iKO^* mice (T2), a chromosome spread assay was conducted in conjunction with meiosis‐indicative markers. First, it was confirmed that hnRNPA2B1 was highly enriched in mid/late pachytene and diplotene cells, and that hnRNPA2B1 was deleted in most spermatocytes in *Hnrnpa2b1^iKO^* mice at T2 (**Figure** **3**A). Through quantitative analysis of distinct spermatocyte subtypes, it was determined that the proportion of late meiotic spermatocytes (pachytene and diplotene) was reduced at T2–T6, while the proportion of early meiotic spermatocytes was elevated in *Hnrnpa2b1^iKO^* mice (Figure 3B), indicating disrupted meiotic prophase I progression.

Chromosomal synapsis and meiotic recombination in spermatocytes are critical processes during meiotic prophase I. γH2AX, a marker for DSB formation in early meiotic spermatocytes, indirectly reflects homologous repair at a later stage. In pachytene spermatocytes from control mice, γH2AX was predominantly localized to the XY chromosome. However, in pachytene spermatocytes from *Hnrnpa2b1^iKO^* mice at T2, the ratios of cells with γH2AX on expanded XY chromosome (≈48.3% in iKO vs ≈3.8% in Ctrl) and retained γH2AX on autosomes (≈69.9% in iKO vs ≈4.9% in Ctrl) were significantly increased (Figure 3C,D), indicating impaired DSB repair in hnRNPA2B1‐deficient pachytene spermatocytes. Similarly, MDC1 (Mediator of DNA damage checkpoint protein 1), which specifically recognizes and binds histone H2AX phosphorylated at ‘Ser‐139’ and serves as a marker of DNA damage, also displayed expanded signals on sex body and retained signal on autosomes (Figure 3E,F). SYCP1, the transverse element of the synaptonemal complex,^[^ ^19^, ^20^ ^]^ was used to detect synapsis. In control pachytene spermatocytes, all autosomes completed synapsis, whereas sex chromosomes underwent partial synapsis in the pseudoautosomal region (PAR). In *Hnrnpa2b1^iKO^* mice at T2, the ratios of XY segregation (≈58.0% in iKO vs ≈1.1% in Ctrl) and autosomal unsynapsis (≈19.6% in iKO vs ≈0.4% in Ctrl) were significantly increased, indicating that hnRNPA2B1 is essential for homologous synapsis (Figure 3G,H).

**Figure 4:**
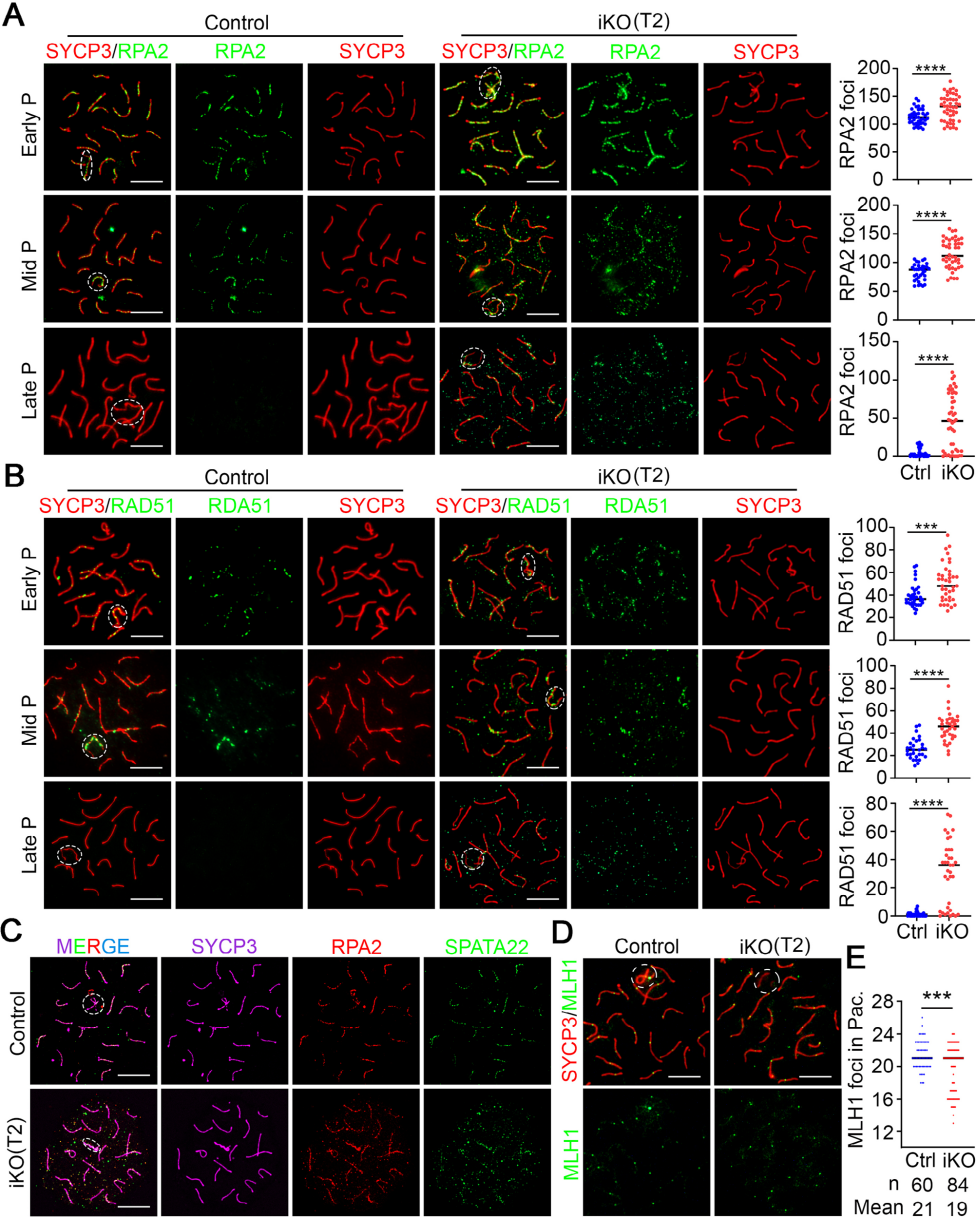
Off‐axis foci of recombination repair‐related proteins in hnRNPA2B1‐depleted pachytene spermatocytes. (A,B) Chromosome spread analysis and quantification of RPA2 (A) and RAD51 (B) in Ctrl and *Hnrnpa2b1* ^iKO^ pachytene spermatocytes at T2. The white dashed lines indicate sex body. Abbreviations: Early P, early pachytene; Mid P, mid pachytene; Late P, late pachytene. Scale bars = 10 µm. The quantified data are presented as mean ± SD. ***p < 0.001, ****p < 0.0001. The spermatocytes were counted from three control and *Hnrnpa2b1* ^iKO^ mice, respectively. (C) Chromosome spread analysis of co‐localization of RPA2 and SPATA22 in control and *Hnrnpa2b1* ^iKO^ pachytene spermatocytes at T2. The white dashed lines indicate sex body. Scale bars = 10 µm. (D,E) Chromosome spread analysis (D) and quantification (E) of MLH1 in Ctrl and *Hnrnpa2b1* ^iKO^ pachytene spermatocytes at T2. The white dashed lines indicate sex body. Scale bars = 10 µm. n, the total number of quantified pachytene spermatocytes. Mean, the average number of MLH1 foci per cell. The quantified data are presented as mean ± SD. ***p < 0.001. Three males per genotype (Ctrl and iKO) were analyzed.

It is evident that the process of homologous recombination is initiated immediately following the formation of DSB, involving the recruitment of ssDNA‐binding proteins, such as RPA2, SPATA22, and MEIOB, and subsequent recombinases, including DMC1 and RAD51, reaching its peak at zygotene spermatocytes and being completed at mid‐pachytene spermatocytes.^[^ ^21^ ^]^ A significant increase in the number of RPA2 and SPATA22 foci was observed in early to late pachytene spermatocytes of *Hnrnpa2b1^iKO^* mice at T2 compared to controls (**Figure** **4**A; Figures S3A and S4A, Supporting Information). Of particular interest was the observation of RPA2 and SPATA22 foci outside the chromosome axes (marked by SYCP3, a component of the lateral synaptonemal complex) in *Hnrnpa2b1^iKO^* spermatocytes (Figure 4A; Figures S3A and S4A, Supporting Information). Other than ssDNA‐binding proteins RPA2 and SPATA22, recombinase foci (RAD51 and DMC1) also exhibited a similar trend in *Hnrnpa2b1^iKO^* pachytene spermatocytes from early to late substage, with localization outside the chromosome axes (Figure 4B; Figures S3B and S4A, Supporting Information). Further co‐staining of RPA2 and SPATA22 revealed that most off‐axis RPA2 signals co‐localized with SPATA22 in *Hnrnpa2b1^iKO^* spermatocytes (Figure 4C). In control mice, homologous recombination‐related proteins (RPA2, SPATA22, RAD51, and DMC1) were consistently observed on chromosome axes. However, the presence of off‐axis foci of these proteins in *Hnrnpa2b1* ^iKO^ pachytene spermatocytes raises the question of whether their recruitment to chromosomal axes is impaired. To address this issue, chromosome spread analyses on leptotene and zygotene spermatocytes were performed, which revealed that almost all foci were located on chromosome axes (Figure S4B–I, Supporting Information). This finding indicates that the recruitment of homologous recombination‐related proteins to the axes was unaffected by the deletion of hnRNPA2B1, and foci outside the chromosome axes were limited to the pachytene stage. Furthermore, the quantification analyses of the foci in early meiotic spermatocytes revealed a significant reduction in zygotene spermatocytes of *Hnrnpa2b1^iKO^* mice at T2 (Figure S4B–I, Supporting Information), suggesting that hnRNPA2B1 can affect homologous recombination in the early meiotic stage.

To rule out the possibility that the pachytene defects observed in *Hnrnpa2b1^iKO^* mice at T2 were caused by earlier zygotene defects, we analyzed mice at additional time points. IF staining for hnRNPA2B1 on testes sections and chromosome spread slides revealed very low knockout efficiency in zygotene spermatocytes of *Hnrnpa2b1^iKO^* mice at T0. In contrast, efficiency was substantially higher at T2 and T6 (Figure S5A–C, Supporting Information). Consistent with the low knockout efficiency at T0, the recombination process marked by RPA2 showed no obvious defects. Conversely, RPA2 foci were significantly reduced in *Hnrnpa2b1^iKO^* mice at T2 and T6 (Figure S5D,E, Supporting Information). Further analysis of the remaining pachytene cells of *Hnrnpa2b1^iKO^* mice at T6 showed that the majority were hnRNPA2B1‐positive (Figure S5F–H, Supporting Information), indicating that early defects arising from the deletion of hnRNPA2B1 could result in zygotene arrest. These results suggest that the defects in pachytene cells are pachytene‐autonomous rather than being a secondary consequence of zygotene defects.

Since the DNA damage response (DDR) functions as a surveillance system for recombination, several classical markers were examined. HORMAD1 and BRCA1, which are essential for ATR recruitment and synapsis^[^ ^22^, ^23^ ^]^ and are located on unsynapsed chromosomes in control mice, were found to be intact in *Hnrnpa2b1^iKO^* mice (Figure S6A,B, Supporting Information). ATR and its activator TOPBP1, which are involved in sensing DNA damage and the second wave of H2AX phosphorylation,^[^ ^24^, ^25^ ^]^ are enriched in the unsynapsed region of the XY chromosomes in the early pachytene stage and in the sex body in the mid‐to‐late pachytene stage in control mice. In *Hnrnpa2b1* ^iKO^ mice, these proteins were always localized to unsynapsed chromosomes in early pachytene, while in mid‐late pachytene spermatocytes, their localization pattern resembled that of γH2AX and MDC1 (Figure S6C,D, Supporting Information). Then, the MRE11‐RAD50‐NBS1 (MRN) complex, which is essential for DNA damage sensing and DSB recombination, was also detected. The results obtained also demonstrated a similar expression pattern to that of γH2AX and MDC1, characterized by persistent signals of the MRN complex on autosomes in *Hnrnpa2b1^iKO^* pachytene spermatocytes (Figure S6E–G, Supporting Information).

In addition, given that the homologous chromosomes exchange genetic information via crossing over in the mid‐pachytene stage, ensuring subsequent chromosome segregation at meiotic metaphase I,^[^ ^21^ ^]^ MLH1, a DNA mismatch repair protein that marks recombination crossover sites, was detected. In control pachytene spermatocytes, a total of ≈21 foci, with at least one per bivalent, were observed within each cell. However, in *Hnrnpa2b1^iKO^* mice at T2, the MLH1 signal was reduced to an average of ≈19 foci (Figure 4D). Of particular note was the significantly reduced foci on sex chromosomes in *Hnrnpa2b1^iKO^* mice (Figure 4E), indicating a disruption of the crossover process in hnRNPA2B1‐depleted pachytene spermatocytes. Collectively, these data demonstrate that hnRNPA2B1 deletion impairs homologous recombination, synapsis, and crossing over in pachytene spermatocytes.

### 2.4 hnRNPA2B1 Affects Pachytene Progression During the First Wave of Spermatogenesis

In order to investigate the role of hnRNPA2B1 in the meiocytes of the first wave, which derive from prospermatogonia rather than spermatogonial stem cells (SSCs), a tamoxifen injection was performed into P13 mice (the time pachytene cells begin to appear) for three consecutive days. Testes at P18, P20, P22, and P24 were then collected for further analysis (Figure S7A, Supporting Information). Histological analysis revealed that no round spermatids were present in the seminiferous tubules of P22 or P24 testis sections in iKO juvenile mice, whereas round spermatids were observed in the majority of tubules in control mice (Figure S7B, Supporting Information). This suggests that meiosis progression is disrupted in iKO juvenile mice. To determine the cause of cell loss in iKO juvenile mice, a TUNEL assay was performed, which revealed a significant increase in apoptotic signals in the middle of seminiferous tubules of iKO juvenile mice, indicating that spermatocytes undergo apoptosis (Figure S7C,D, Supporting Information). This finding was further corroborated by the observation that co‐staining of hnRNPA2B1 and Cleaved‐Caspase 3 (an apoptosis marker) revealing that apoptotic spermatocytes in iKO juvenile testes were predominantly hnRNPA2B1‐negative, thereby confirming that hnRNPA2B1‐depleted cells undergo apoptosis (Figure S7E, Supporting Information). Furthermore, H1T and SYCP3 were utilized as markers to identify mid‐late pachytene spermatocytes and diplotene spermatocytes in P20 control and iKO juvenile testis sections. The results obtained revealed a significant reduction in the number of mid‐late pachytene or diplotene spermatocytes in iKO juvenile mice (≈13 in Ctrl and ≈6 in iKO). Notably, in some seminiferous tubules, no H1T‐positive cells were observed (Figure S7F,G, Supporting Information). Together, these observations suggest that hnRNPA2B1 plays a conserved role in regulating late meiotic progression during both the first wave and steady‐state spermatogenesis.

### 2.5 hnRNPA2B1 Deletion Leads to Dramatic Alteration in Transcriptome and Proteome

**Figure 5:**
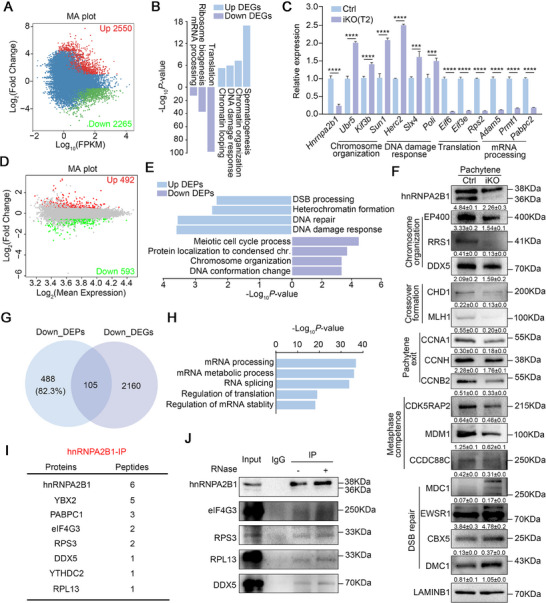
Dramatic transcriptome and proteome alterations exist in *Hnrnpa2b1* ^iKO^ pachytene spermatocytes. (A) MA plot of the transcriptome in pachytene spermatocytes from Ctrl and *Hnrnpa2b1* ^iKO^ mice at T2. The differentially expressed (up‐ or down‐regulated) genes are shown as red or green dots. (B) GO terms of up‐ or down‐regulated genes in *Hnrnpa2b1* ^iKO^ pachytene spermatocytes. (C) RT‐qPCR analysis of selected DEGs in *Hnrnpa2b1* ^iKO^ pachytene spermatocytes. The quantified data are presented as mean ± SD. ***p < 0.001, ****p < 0.0001. Four males per genotype (Ctrl and iKO) were analyzed. (D) MA plot of proteome in pachytene spermatocytes of Ctrl and *Hnrnpa2b1* ^iKO^ mice at T2. The differentially expressed (up‐ or down‐regulated) proteins are shown as red or green dots. (E) GO terms of up‐ and down‐regulated proteins in *Hnrnpa2b1* ^iKO^ pachytene spermatocytes. (F) Western blot and quantification for verification of selected proteins in Ctrl and *Hnrnpa2b1* ^iKO^ pachytene spermatocytes. LAMIN B1 serves as loading control. Quantifications of hnRNPA2B1 account for both two bands. The quantified data are presented as mean ± SD. *n* = 3 mice. (G) Overlap of downregulated DEGs and downregulated DEPs. (H) GO terms of potential interactors of hnRNPA2B1 in P15 testis. (I) List of some potential interactors identified by IP‐MS. Proteins detected in the IP group but not in the IgG group were considered interacting proteins. (J) Co‐IP to validate the RNA‐independent interactions between hnRNPA2B1 and eIF4G3, RPS3, RPL13, and DDX5.

To elucidate the molecular defect present in *Hnrnpa2b1^iKO^* pachytene spermatocytes, pachytene cells were isolated from both control and iKO adult mice at T2 for comprehensive RNA‐sequencing and proteomic analyses. The purity of the isolated spermatocytes was confirmed by SYCP3/γH2AX co‐staining (Figure S8A, Supporting Information), and only samples with a purity exceeding 80% were selected for sequencing and subsequent validation. The subsequent transcriptomic analysis identified 2550 up‐regulated and 2265 down‐regulated genes (referred to as Up DEGs (differentially expressed genes) and Down DEGs, respectively) in *Hnrnpa2b1^iKO^* pachytene spermatocytes (**Figure** **5**A and Table S1, Supporting Information). Gene Ontology (GO) analysis revealed that Up DEGs were associated with spermatogenesis, chromatin organization, DNA damage response, and chromatin looping, while Down DEGs were related to translation, ribosome biogenesis, and mRNA processing (Figure 5B). The validation of several DEGs via RT‐qPCR confirmed that genes involved in chromosome organization (*Ubr5*, *Kif3b*, *Sun1*) and DNA damage response (*Herc2*, *Slx4*, *Poli*) were significantly upregulated, whereas those related to translation (*Eif6*, *Eif3e*, *Rps2*) and mRNA processing (*Adam5*, *Prmt1*, *Pabpc2*) were downregulated (Figure 5C; Figure S8B, Supporting Information). Proteomic profiling identified 1085 differentially expressed proteins (DEPs), with 492 up‐regulated and 593 down‐regulated (Figure 5D and Table S2, Supporting Information). GO term analysis indicated that the DEPs that were found to be expressed at elevated levels were associated with DSB processing, heterochromatin formation, DNA repair, and DNA damage response, while the DEPs that were found to be expressed at reduced levels were related to meiotic cell cycle process, protein localization to condensed chromosomes, chromosome organization, and DNA conformation change (Figure 5E). Specifically, elevated levels of DNA damage response proteins (MDC1, EWSR1, CBX5, DMC1, MSH4, M1AP, TERB2, TP53BP1) were observed, while reduced levels of proteins associated with chromosome organization (EP400, RRS1, DDX5, CHD1, RIF1, CHRAC1, UBR2) and pachytene exit (CCNA1, CCNB2, CCNB1, CCNB1IP1, CCNT2, CCNH) were noted (Table S2, Supporting Information). To validate these findings, a Western blot assay was performed, which confirmed a significant reduction in protein levels associated with chromosome organization (EP400, RRS1, DDX5), crossover formation (CHD1, MLH1), pachytene exit (CCNA1, CCNH, CCNB2), and metaphase competence (CDK5RAP2, MDM1, CCDC88C) in hnRNPA2B1‐depleted pachytene spermatocytes, while the protein levels involved in DSB repair (MDC1, EWSR1, CBX5, DMC1) were increased (Figure 5F). A notable observation was that the majority of genes exhibiting down DEPs (82.3%) or up DEPs (83.7%) demonstrated no corresponding decrease or increase in DEGs (Figure 5G; Figure S8C, Supporting Information), suggesting that hnRNPA2B1 may play a crucial role in post‐transcriptional regulation to modulate protein levels of meiosis‐related genes.

The pronounced alterations observed in both the transcriptome and proteome of *Hnrnpa2b1^iKO^* mice prompted us to investigate potential interactors of hnRNPA2B1. Consequently, an analysis of IP‐MS data obtained from P15 testes was conducted, and the GO terms of these interactors were found to be closely related to mRNA processing, mRNA metabolism, RNA splicing, regulation of translation, and regulation of mRNA stability (Figure 5H). Subsequent analysis of the IP‐MS dataset identified YBX2, PABPC1, eIF4G3, RPS3, DDX5, YTHDC2, and RPL13 as the most probable interactors (Figure 5I). Subsequent co‐immunoprecipitation (Co‐IP) assay on pachytene spermatocytes was conducted for validation purposes, which demonstrated that hnRNPA2B1 does indeed interact with translation factors (e.g., eIF4G3, RPS3, RPL13) and mRNA processing regulators (DDX5) in an RNA‐independent manner (Figure 5J). Taken together, these results of the sequencing data and the validation experiments suggest that hnRNPA2B1 interacts with regulators of mRNA processing and translation to modulate key meiosis genes, thereby controlling pachytene progression.

### 2.6 hnRNPA2B1 Targets Several Meiotic Genes and Recognizes Their m^6^A Sites

**Figure 6:**
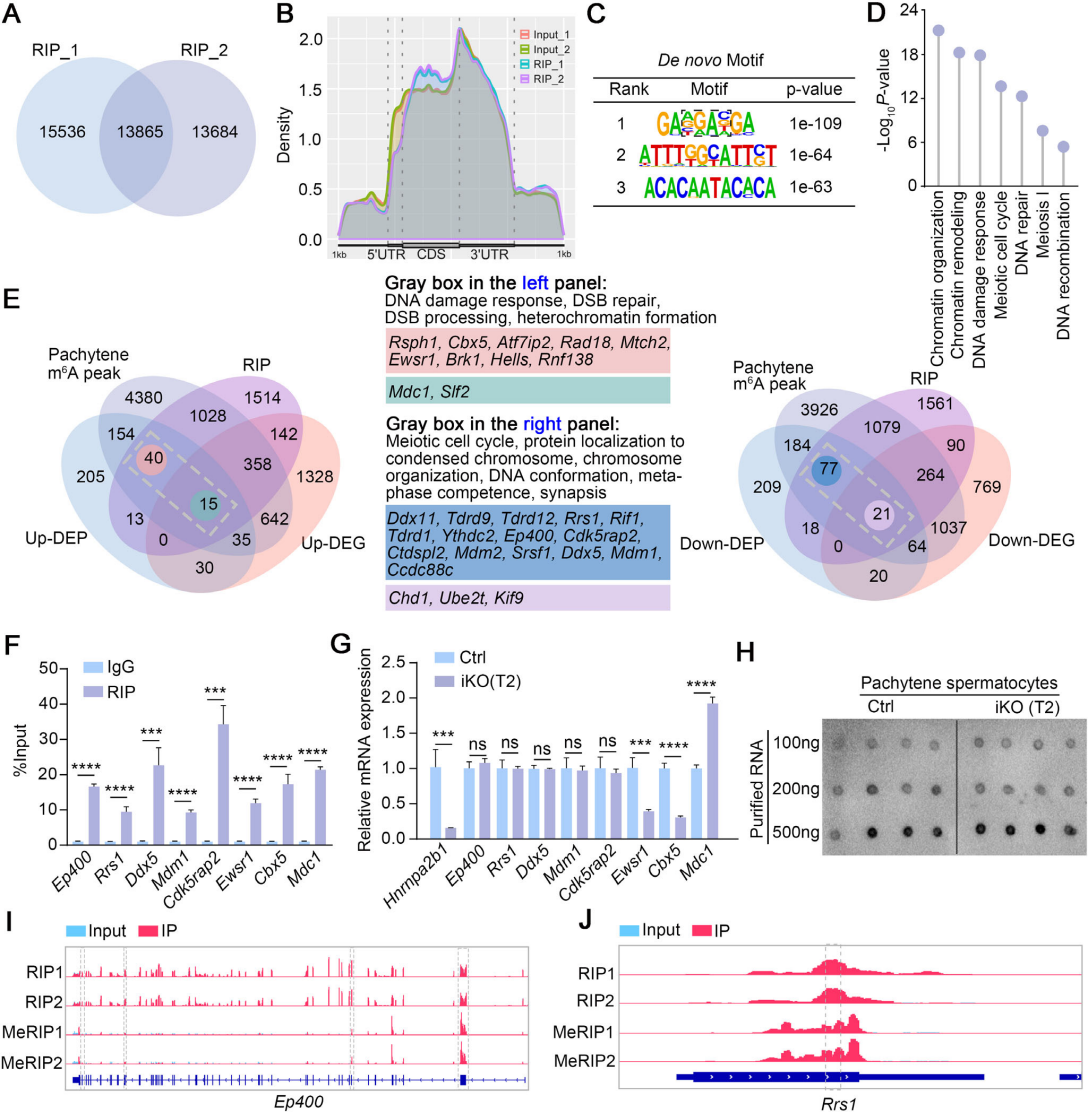
hnRNPA2B1 targets transcripts to regulate their expression in an m^6^A‐dependent manner in pachytene spermatocytes. (A) Peaks identified by RIP‐seq in two replicates. (B) Peak distribution of hnRNPA2B1 determined by RIP‐seq. (C) *De novo* motif analysis determined by HOMER in RIP‐seq. (D) GO terms of hnRNPA2B1 targets in pachytene spermatocytes. (E) Overlap analysis of RIP‐seq, MeRIP‐seq, RNA‐seq, and proteomics data. GO terms of the genes in gray boxes in the left and right images are listed in the middle panel. The middle panel was divided into four gene sets: Pink panel, inconsistent trend in mRNA and protein levels in the left graph; Green panel, consistent trend in mRNA and protein levels in the left graph; Blue panel, inconsistent trends in mRNA and protein levels in the right graph; Purple panel, consistent trends in mRNA and protein levels in the right graph. (F) RIP‐qPCR of selected genes identified by RIP‐seq in pachytene spermatocytes. Quantified data are presented as mean ± SD. ***p < 0.001, ****p < 0.0001. Four males per genotype (Ctrl and iKO) were analyzed. (G) RT‐qPCR to confirm mRNA levels of selected target genes in pachytene spermatocytes of Ctrl and *Hnrnpa2b1* ^iKO^ mice at T2. Quantified data are presented as mean ± SD. ***p < 0.001, ****p < 0.0001. ns, not significant. Four males per genotype (Ctrl and iKO) were analyzed. (H) m^6^A dot plot of Ctrl and *Hnrnpa2b1* ^iKO^ pachytene spermatocytes. (I,J) IGV plots of *Ep400* and *Rrs1* identified by RIP‐seq and MeRIP‐seq.

To identify direct targets of hnRNPA2B1, RNA immunoprecipitation sequencing (RIP‐seq) was performed on purified pachytene spermatocytes. This analysis yielded a total of 3110 genes that were identified as direct targets of hnRNPA2B1, with 13865 overlapping peaks observed in two replicates (**Figure** **6**A and Table S3, Supporting Information). Subsequent analysis revealed that these peaks were predominantly enriched in the CDS/3’UTR regions (Figure 6B). *De novo* motif analysis indicated that hnRNPA2B1 primarily recognizes the RGAC motif (Figure 6C), which is a classical m^6^A motif. GO analysis of these targets highlighted associations with chromatin organization, chromatin remodeling, DNA damage response, meiotic cell cycle, meiosis I, and DNA recombination (Figure 6D).

Given that hnRNPA2B1 is a classical m^6^A reader, the present study combined the sequencing data with other published m^6^A data^[^ ^3^ ^]^ was analyzed. The analysis of RIP‐seq, up‐DEPs, up‐DEGs, and m^6^A data identified 55 up‐regulated proteins whose RNAs were directly targeted by hnRNPA2B1 and underwent m^6^A modification. Of these, 15 (27.3%) exhibited up‐regulated mRNA levels, while 40 (72.7%) showed down‐regulated or unchanged levels. Notably, among these 55 genes, several genes related to DNA damage response, DSB repair, DSB processing, and heterochromatin formation, including *Rsph1*, *Cbx5*, *Atf7ip2*, *Rad18*, *Mtch2*, *Ewsr1*, *Brk1*, *Hells*, *Rnf138*, *Mdc1*, and *Slf2*, were identified. Similarly, analysis of RIP‐seq, down‐DEPs, down‐DEGs, and m^6^A data identified 98 down‐regulated proteins whose transcripts were m^6^A‐modified and directly targeted by hnRNPA2B1. Among these, 21 (21.4%) showed down‐regulated mRNA levels, while 77 (78.6%) showed up‐regulated or unchanged levels. Among these 98 genes, several genes associated with the meiotic cell cycle, protein localization to condensed chromosomes, chromosome organization, DNA conformation, metaphase competence, and synapsis, including *Ddx11*, *Tdrd9*, *Tdrd12*, *Rrs1*, *Rif1*, *Tdrd1*, *Ythdc2*, *Ep400*, *Cdk5rap2*, *Ctdspl2*, *Mdm2*, *Srsf1*, *Ddx5*, *Mdm1*, *Ccdc88c, Chd1, Ube2t, Kif9*, were identified (Figure 6E). Further RIP‐qPCR analysis confirmed that *Ep400*, *Rrs1*, *Ddx5*, *Mdm1*, *Cdk5rap2*, *Ewsr1*, *Cbx5* and *Mdc1* were targeted by hnRNPA2B1 (Figure 6F). Concurrently, the mRNA levels of *Ep400*, *Rrs1*, *Ddx5*, *Mdm1*, and *Cdk5rap2* remained unchanged, *Ewsr1*, *Cbx5* were reduced, while that of *Mdc1* was increased in hnRNPA2B1‐depleted pachytene spermatocytes, a finding that was consistent with the RNA‐seq data (Figure 6G).

To investigate the relationship between hnRNPA2B1 and other m^6^A readers, Co‐IP assays were performed using isolated pachytene spermatocytes. The results revealed RNA‐independent interactions between hnRNPA2B1 and YTHDC2, YTHDC1 and IGF2BP3. No interaction was observed between hnRNPA2B1 and hnRNPC, YTHDF1, YTHDF3, or FMR1 (Figure S8D, Supporting Information). Among three interacting m^6^A readers, the phenotype of late pachytene apoptosis in *Ythdc2* ^iKO^ mice was similar to that in *Hnrnpa2b1* ^iKO^ mice,^[^ ^8^ ^]^ thus promoting a comparison of DEGs between the two mutant mice. The results demonstrated that a mere 348 (13.6%) and 504 (22.3%) genes of up‐DEGs and down‐DEGs, respectively, exhibited congruent trends in *Ythdc2* ^iKO^ pachytene spermatocytes (Figure S8E, Supporting Information). Furthermore, a mere 121 genes (3.9%)^[^ ^26^ ^]^ or 9 genes (0.3%)^[^ ^27^ ^]^ of hnRNPA2B1‐targeted genes were also targeted by YTHDC2 (Figure S8F, Supporting Information), thereby indicating a weaker relationship between the m^6^A readers hnRNPA2B1 and YTHDC2‐directed pachytene defect. Of note, while the total m^6^A level remained unaltered between the control and hnRNPA2B1‐depleted pachytene spermatocytes (Figure 6H), gene plots of selected genes *Ep400* and *Rrs1*, which are crucial for chromosome organization and remodeling, exhibited overlapping peaks in their exons from RIP‐seq and MeRIP‐seq analyses (Figure 6I,J), indicating that hnRNPA2B1‐mediated m^6^A recognition may regulate specific transcripts. Together, these findings suggest that hnRNPA2B1 could directly regulate the expression of critical meiosis genes, possibly in a m^6^A‐dependent manner, to monitor pachytene progression.

### 2.7 hnRNPA2B1‐Mediated m^6^A Recognition Enhances Expression of Chromosome Organization‐Related Genes

**Figure 7:**
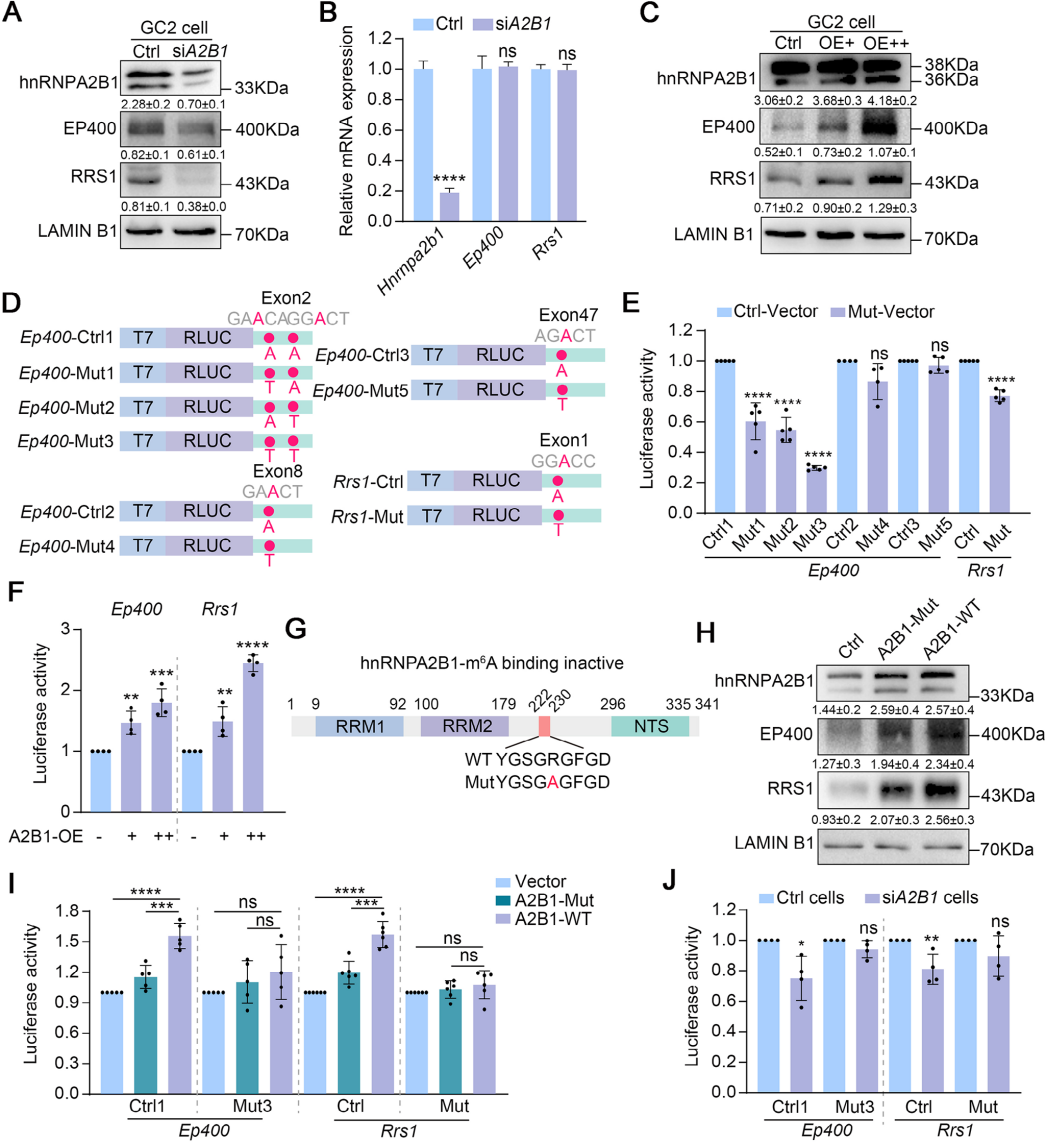
hnRNPA2B1‐mediated m^6^A recognition promotes expression of genes related to chromosome organization. (A) Western blot analysis of hnRNPA2B1, EP400, and RRS1 in Ctrl and si*A2B1* GC2 cells. LAMIN B1 is used as loading control. Quantifications of hnRNPA2B1 account for both two bands. The quantified data are presented as mean ± SD. Three replicates per group were analyzed. (B) RT‐qPCR to confirm the mRNA levels of *Hnrnpa2b1*, *Ep400*, and *Rrs1* in Ctrl and si*A2B1* GC2 cells. Quantified data are presented as mean ± SD. ****p < 0.0001. ns, not significant. Four replicates per group (Ctrl and si*A2B1*) were analyzed. (C) Western blot analysis of hnRNPA2B1, EP400, and RRS1 in Ctrl and hnRNPA2B1‐overexpressing GC2 cells. LAMIN B1 is used as loading control. Abbreviations: OE, hnRNPA2B1 overexpression. + and ++ indicate different levels of hnRNPA2B1 overexpression. Quantifications of hnRNPA2B1 account for both two bands. The quantified data are presented as mean ± SD. Three replicates per group were analyzed. (D) Construction of control and mutant luciferase reporters of *Ep400* and *Rrs1*. (E) Luciferase activity of control and mutant luciferase vectors in GC2 cells. Quantified data are presented as mean ± SD. ****p < 0.0001. ns, not significant. At least four replicates per group were analyzed. (F) Luciferase activity of control luciferase vectors (*Ep400*‐Ctrl1 and *Rrs1*‐Ctrl) with hnRNPA2B1 overexpression. Quantified data are presented as mean ± SD. **p < 0.01, ***p < 0.001, ****p < 0.0001. Four replicates per group were analyzed. (G) Construction of control and m^6^A binding inactive hnRNPA2B1 vectors. (H) Western blot assay of hnRNPA2B1, EP400, and RRS1 in CMV‐Myc, Myc‐hnRNPA2B1, and Myc‐hnRNPA2B1‐Mut GC2 cells. Quantifications of hnRNPA2B1 account for both two bands. The quantified data are presented as mean ± SD. Three replicates per group were analyzed. (I) Luciferase activity of control and mutant vectors in GC2 cells co‐expressing CMV‐Myc, Myc‐hnRNPA2B1, or Myc‐hnRNPA2B1‐Mut vectors. Quantified data are presented as mean ± SD. ***p < 0.001, ****p < 0.0001. ns, not significant. At least five replicates per group were analyzed. (J) Luciferase activity of control and mutant vectors in Ctrl and si*A2B1* cells. Quantified data are presented as mean ± SD. *p < 0.05, **p < 0.01. ns, not significant. Four replicates per group were analyzed.

To ascertain the exact relationship between hnRNPA2B1‐mediated m^6^A recognition and gene expression, an RNAi approach was employed in vitro. Treatment of GC2 cells (a mouse spermatocyte cell line) with siRNA resulted in a significantly reduction in hnRNPA2B1 levels, both at the protein and mRNA levels (**Figure** **7**A,B), thereby indicating effective knockdown. Consistent with in vivo results, protein levels of EP400 and RRS1 decreased significantly in knockdown GC2 cells (Figure 7A), whereas mRNA levels remained unchanged (Figure 7B). Conversely, the protein levels of EP400 and RRS1 exhibited a marked increase in response to hnRNPA2B1 overexpression (referred to as OE+ or OE++) (Figure 7C). This finding suggests that hnRNPA2B1 may also regulate the expression of *Ep400* and *Rrs1* in vitro. To further investigate this, mutant CDS/3’UTR luciferase reporters were designed and constructed by replacing the specific adenosine (A) in the m^6^A motif with threonine (T) based on the control luciferase reporters (Figure 7D). The results of dual luciferase assays indicate that the selected m^6^A sites in exon 2 of *Ep400* and exon 1 of *Rrs1* have the capacity to affect luciferase activity, while the others do not (Figure 7E). To further investigate the role of hnRNPA2B1 in regulating luciferase activity, *Ep400*‐Ctrl1 and *Rrs1*‐Ctrl1 vectors were co‐transfected with varying amounts of hnRNPA2B1 into GC2 cells. The results obtained from this experiment demonstrated that a greater elevated level of hnRNPA2B1 was more capable of promoting luciferase activity to a greater extent than a lower level, in both the *Ep400* and *Rrs1* groups (Figure 7F). This finding suggests that hnRNPA2B1 exerts a regulatory influence on the expression of EP400 and RRS1.

In addition, to assess whether the m^6^A recognition function of hnRNPA2B1 is implicated in the regulation of EP400 and RRS1 expression, wild‐type (*A2b1*‐WT) and m^6^A binding inactive hnRNPA2B1 (*A2b1*‐Mut) overexpressing vectors^[^ ^28^, ^29^ ^]^ were constructed and a transfection was performed (Figure 7G). Western blot analysis revealed a more significant increase of EP400 and RRS1 proteins in the *A2b1*‐WT group compared to the *A2b1*‐Mut group (Figure 7H), suggesting that hnRNPA2B1 regulates the expression of EP400 and RRS1 through m^6^A recognition. The luciferase assay further confirmed that hnRNPA2B1 was unable to enhance luciferase activity in reporters with mutant m^6^A sites (Mut3 in *Ep400* and Mut in *Rrs1*), and that the increase in luciferase activity was more pronounced in *A2b1*‐WT than *A2b1*‐Mut (Figure 7I). Furthermore, the luciferase assay of control and si*A2B1* cells confirmed that the knockout of hnRNPA2B1 significantly reduced luciferase activity only in the control luciferase reporters, and not in the mutant ones (Figure 7J). Taken together, these findings emphasize the pivotal role of hnRNPA2B1‐mediated m^6^A recognition in promoting the expression of genes that are essential for chromosome organization and remodeling.

### 2.8 hnRNPA2B1 Deficiency does not Affect MSCI, Telomere Morphology, and Cohesin Subunit Localization in Pachytene Spermatocytes

It has been demonstrated by preceding studies that impaired meiotic sex chromosome inactivation (MSCI) results in pachytene apoptosis and meiosis arrest.^[^ ^30^ ^]^ In order to ascertain whether MSCI is affected in iKO spermatocytes, an analysis of the transcriptome level on each chromosome was conducted. The resultant data did not reveal an obvious increase in gene expression on sex chromosomes like other MSCI‐defective mutants^[^ ^4^, ^31^, ^32^, ^33^ ^]^ (Figure S8G, Supporting Information). However, the subsequent analysis of transcripts on autosomes, X and Y chromosome, revealed a more pronounced increase of Log_2_FC on X chromosome than autosomes (Figure S8H, Supporting Information), indicating that the transcription in X chromosome was more affected than autosomes. To experimentally confirm the hypothesis that MSCI may be defective, a chromosome spread assay was performed, which revealed no accumulation of RNA POL II and H3K9AC (markers for MSCI) in the sex body of iKO pachytene spermatocytes at T2 (Figure S8I,J, Supporting Information). This suggests that MSCI was intact in hnRNPA2B1‐deleted pachytene cells and that pachytene apoptosis was not due to MSCI checkpoint‐mediated cell elimination.

Furthermore, the sequencing data revealed a reduction in proteins associated with microtubule organization and bundle formation, including CCNB2, MARK2, PCM1, NDC80, SPC25, MEIKIN, and CCNB1IP1, amongst others (Table S2, Supporting Information). Evidently, the Shelterin complex (the core of the telomere complex), the LINC complex, TERB1, TERB2, and MAJIN, as well as microtubules, form a machine that directs rapid chromosome movement along the nuclear envelope (NE) at telomeres. It is important to note that the correct structure and behavior of telomeres is critical during these processes.^[^ ^8^, ^34^, ^35^, ^36^, ^37^, ^38^ ^]^ Given that hnRNPA2B1 has been previously reported to be implicated in telomere maintenance and DNA repair in somatic cells,^[^ ^39^, ^40^ ^]^ we sought to investigate whether hnRNPA2B1 depletion affects telomere structure and behavior. To this end, TRF1, TRF2, and POT1, subunits of shelterin complex, were labeled, as they localize to chromosome ends to protect them from DNA damage response (DDR) attack and are involved in anchoring telomeres to the NE, on chromosome spread slides. Notably, no significant morphological alterations were observed in iKO pachytene spermatocytes. In both control and iKO pachytene cells, TRF1, TRF2, and POT1 were predominantly localized at chromosome ends, appearing as distinct dot‐like signals (Figure S9A–C, Supporting Information).

Furthermore, since the sequencing data demonstrated a dysregulation of the transcriptome and proteome involved in chromosome organization and assembly, along with mislocalization of recombination proteins in iKO pachytene spermatocytes identified by chromosome spread assay, the localization of cohesin factors SMC3, REC8, and RAD21, three canonical cohesin components that are critical for chromosome organization and assembly,^[^ ^41^ ^]^ was examined. The results of the immunostaining demonstrated that none of these cohesin factors exhibited any noticeable mislocalization or defects in iKO pachytene spermatocytes (Figure S9D–F, Supporting Information). Altogether, these observations suggest that the pachytene apoptosis observed in iKO mice is unlikely to be directly associated with MSCI failure, telomere dysfunction, or cohesin‐related defects.

## 3 Discussion

In our previous study, it was reported that the global knockout of *Hnrnpa2b1* in mice results in a Sertoli cell‐only phenotype,^[^ ^17^ ^]^ thereby hindering the investigation of the function of hnRNPA2B1 in germ cell development. In this study, a tamoxifen‐induced inactivation strategy was employed to uncover a novel function of hnRNPA2B1 in pachytene progression during meiotic prophase I. This discovery aligns with the observed enrichment of hnRNPA2B1 in pachytene spermatocytes, underscoring its essential role in late meiotic prophase I and highlighting its significance in pachytene progression. Specifically, depletion of hnRNPA2B1 at the pachytene stage has been shown to result in extensive dysregulation of the transcriptome and proteome, leading to the arrest of *Hnrnpa2b1* ^iKO^ cells at late pachytene stage.

In the present study, it was observed that *Hnrnpa2b1* ^iKO^ meiotic cells undergo apoptosis at the late pachytene stage (VIII–X), accompanied by significant alterations in the transcriptome and proteome. This observation is consistent with a previous study, which demonstrated that tamoxifen‐induced *Ythdc2* deletion leads to late pachytene apoptosis, possibly due to dramatic transcriptomic changes or overexpressed X‐linked genes. In line with the observations made in *Ythdc2* ^iKO^ pachytene spermatocytes,^[^ ^8^ ^]^ our subsequent analysis, which included an examination of the transcriptome and immunofluorescence staining, revealed a more affected transcript level on the X chromosome of *Hnrnpa2b1* ^iKO^ mice. However, no abnormality of RNA POL II signal was detected in *Hnrnpa2b1* ^iKO^ pachytene cells, thus ruling out impaired MSCI as a contributing factor and supporting a proposal of late pachytene checkpoint. It is noteworthy that, akin to the sequencing data from *Ythdc2* ^iKO^ pachytene cells, our sequencing data also unveiled numerous genes implicated in microtubule organization and bundle formation. Despite the established function of telomeres in nuclear envelope attachment and chromosome movement,^[^ ^34^, ^35^, ^36^, ^37^, ^38^ ^]^ our chromosome spread assays did not reveal any overt telomere‐related defects, such as aberrations in telomere‐binding proteins, chromosome fusion, or TC pachytene, which were observed in other telomere‐defective mutants.^[^ ^8^, ^42^, ^43^, ^44^ ^]^ This suggests that the pachytene defects directed by hnRNPA2B1 may not be attributable to telomere dysfunction. Furthermore, although YTHDC2 and hnRNPA2B1 exhibit RNase‐independent interactions, the present study revealed a distinct relationship between m^6^A modification and gene expression in hnRNPA2B1‐deficient pachytene spermatocytes, contrasting with *Ythdc2* ^iKO^ cells where minimal correlation between DEGs and m^6^A modification was observed.^[^ ^8^ ^]^ Thus, the current finding indicates that hnRNPA2B1 functions through a distinct mechanism in late pachytene spermatocytes compared to YTHDC2.

It has been established through previous studies that recombination events predominantly occur within chromatin loops,^[^ ^45^ ^]^ which are precisely regulated by various structural and regulatory proteins, including SMC3, PDS5, CTCF, WAPL etc.^[^ ^46^, ^47^ ^]^ Indeed, the chromosome spread assay in the present study revealed an abnormal spatial localization of recombination proteins in hnRNPA2B1‐depleted pachytene spermatocytes, that is, far away from the chromosome axes in *Hnrnpa2b1^iKO^* mice. The off‐axis signals of these recombination proteins were exclusively detected in pachytene cells and were absent in leptotene or zygotene stages, indicating that their recruitment to the chromosomal axis remains intact. However, colocalization of recombination proteins RPA2 and SPATA22 was observed in *Hnrnpa2b1* ^iKO^ pachytene cells, suggesting that these proteins are located at a specific off‐axis structure or still form complex after detaching from axes. Several possibilities may account for this observation: i) Defects in axis‐loop structure. DSBs occurred on chromatin loops, and recombination occurred when the loops tethered to the chromosome axis.^[^ ^45^ ^]^ The highly expressed hnRNPA2B1 in pachytene spermatocytes may participate in stabilizing axis‐loop structure in this stage via regulating genes related to chromosome organization and looping. In the absence of hnRNPA2B1, some axis‐loop structure dissociated, and the ssDNA, still wrapped by recombination‐related proteins was positioned outside the axis along with the loop. ii) Defects in tethering to axes. It is hypothesized that the function of hnRNPA2B1 is to stabilize the attachment of them via its effect on the chromosome microenvironment or the regulation of auxiliary factors in pachytene spermatocytes. This is due to the fact that the MEIOB/SPATA22 complex cooperates with RPA to form a compacted mixed MEIOB/SPATA22/RPA/ssDNA complex.^[^ ^48^ ^]^ In the absence of hnRNPA2B1, this mixed functional complex was detached from the axes. iii) Defects in nuclear export. hnRNPA2B1 may govern the nuclear export of recombination‐related proteins. Under normal conditions, these proteins are removed from the axes during pachytene stage, exported from the nucleus and degraded. However, hnRNPA2B1 deficiency may disrupt this export process, resulting in the accumulation of these proteins in the off‐axis region. Moreover, in early meiotic cells with hnRNPA2B1 deletion, defective DSB repair leads to zygotene arrest (iKO mice at T6), aligning with an established understanding that DSB repair‐defective cells will arrest at mid‐pachytene or even earlier.^[^ ^49^, ^50^, ^51^, ^52^ ^]^ Considering this, along with our analyses of zygotene cells at T0 and the ≈7‐day duration of pachytene,^[^ ^53^ ^]^ we conclude that the defects observed in the pachytene cells of *Hnrnpa2b1* ^iKO^ mice at T2 are pachytene‐autonomous, rather than being a secondary consequence of zygotene defects.

It is noteworthy that our sequencing data on pachytene cells primarily identified differentially expressed genes associated with chromosomal organization and remodeling, which has led to a focused investigation on this regulatory mechanism. The RNA‐seq data revealed a significant upregulation of transcripts associated with meiotic progression, particularly in terms of chromatin organization and assembly. However, proteomic analysis of pachytene spermatocytes showed a discordant trend, with reduced levels of proteins associated with chromosome organization, crossover formation, pachytene exit, and metaphase competence, and increased levels of proteins associated with DSB repair in *Hnrnpa2b1* ^iKO^ pachytene spermatocytes, prompting us to further investigate post‐transcriptional regulation. In particular, our IP‐MS analysis of juvenile testes revealed a cohort of hnRNPA2B1 interactors predominantly involved in mRNA processing and translation, and the Co‐IP assay using pachytene spermatocytes validated the RNA‐independent interactions between hnRNPA2B1 and eIF4G3, RPS3, RPL13, DDX5 and YTHDC2, potentially explaining the profound changes in mRNA and protein expression profiles upon hnRNPA2B1 depletion. Nevertheless, the key interactions essential for pachytene progression remain enigmatic and merit further in‐depth investigation. Furthermore, RIP‐seq identified multiple transcripts as targets of hnRNPA2B1, which are related to chromatin organization and remodeling, DNA damage response, and meiotic cell cycle etc. Notably, an integrative analysis of RNA‐seq, proteomics, RIP‐seq, and public MeRIP‐seq revealed a list of genes, highlighting the essential role of hnRNPA2B1‐mediated m^6^A recognition in regulating gene expression, especially those related to chromosome organization and meiotic cycle. However, further research is required to elucidate the precise regulatory mechanism by which hnRNPA2B1‐mediated m^6^A recognition controls meiosis progression.

In this study, the primarily focus was on post‐transcriptional regulation, with a particular emphasis on the alterations in protein levels. Intriguingly, our RNA‐seq data highlighted that the up‐DEGs were intimately associated with chromosome organization, prompting an inquiry into the mechanisms by which hnRNPA2B1 modulates transcriptional activity. A recent investigation has revealed that following a DSB, SIRT6‐mediated deacetylation of hnRNPA2B1 promotes its dissociation from damaged chromosomes and diminishes RNAPII complex assembly, thereby repressing transcription and safeguarding genome integrity.^[^ ^54^ ^]^ It is plausible that in the absence of hnRNPA2B1, RNAPII accumulates and local transcriptional repression is compromised in pachytene spermatocytes, or alternatively, hnRNPA2B1 may modulate the levels of certain transcription factors or chaperons to indirectly influence the transcriptional process. Furthermore, our recent research has demonstrated that the m^6^A reader hnRNPC (a member of the hnRNP family) governs alternative splicing in differentiating spermatogonia through m^6^A recognition.^[^ ^16^ ^]^ Consequently, it remains to be elucidated whether the observed changes in alternative splicing processes in our RNA‐seq data are also attributable to the m^6^A‐related function of hnRNPA2B1, a hypothesis that beckons further exploration. Beyond the role of hnRNPA2B1 in transcriptional and post‐transcriptional regulation, the observed “expanded sex body” phenotype in *Hnrnpa2b1* ^iKO^ pachytene spermatocytes presents a compelling phenotype warranting further investigation. The formation and dynamics of the sex body may involve liquid‐liquid phase separation (LLPS), which has been shown to concentrate DNA damage response (DDR) factors, chromatin remodelers, and epigenetic modifiers. Concurrently, it excludes non‐essential components, thereby establishing a phase‐separated microenvironment that facilitates functional specialization. During the progression from the early to mid‐late pachytene stages, the sex body undergoes a liquid‐to‐gel transition. Critical proteins, such as MDC1, which contains an IDR, exhibit physicochemical properties conducive to LLPS.^[^ ^55^ ^]^ Given that hnRNPA2B1 is a known regulator of phase separation in spermatogenesis^[^ ^56^ ^]^ and other biological process,^[^ ^57^ ^]^ it is expected to modulate LLPS dynamics, thereby affecting sex body formation during the pachytene stage.

In addition, it should be noted that the utilization of a tamoxifen‐induced knockout mouse model enabled the circumvention of pre‐meiotic or early meiotic arrest, thereby facilitating a stage‐specific investigation. However, as previously highlighted in our research using the *Ddx4*‐Cre^ERT2^ model,^[^ ^4^ ^]^ the knockout efficiency exhibited significant variation among mice and across different time points. The temporal knockout pattern that was observed in the present study is likely to reflect the unique properties of the *Ddx4*‐Cre^ERT2^ system. It is evident that a number of factors may contribute to the earlier and more efficient recombination in pachytene spermatocytes. First, peak DDX4 expression during the pachytene stage provides higher basal Cre^ERT2^ levels.^[^ ^58^ ^]^ Second, enhanced transcriptional activity in pachytene cells may facilitate nuclear translocation of Cre^ERT2^ efficiently.^[^ ^59^, ^60^ ^]^ Moreover, the tamoxifen injection affected all DDX4‐expressing cells, thereby complicating the identification of direct mechanisms underlying cell death. Given the continuous nature of spermatogenesis, the early time point (T2) for meiosis analysis in this study was selected, at a time when the deletion efficiency was suboptimal, in order to avoid widespread disruption of spermatogenesis. These limitations constrained our ability to explore certain subtle yet critical mechanisms. Notwithstanding these limitations, the present data provide robust support for the conclusion that hnRNPA2B1 is indispensable for late meiotic progression in male mice.

## 4 Conclusion

**Figure 8:**
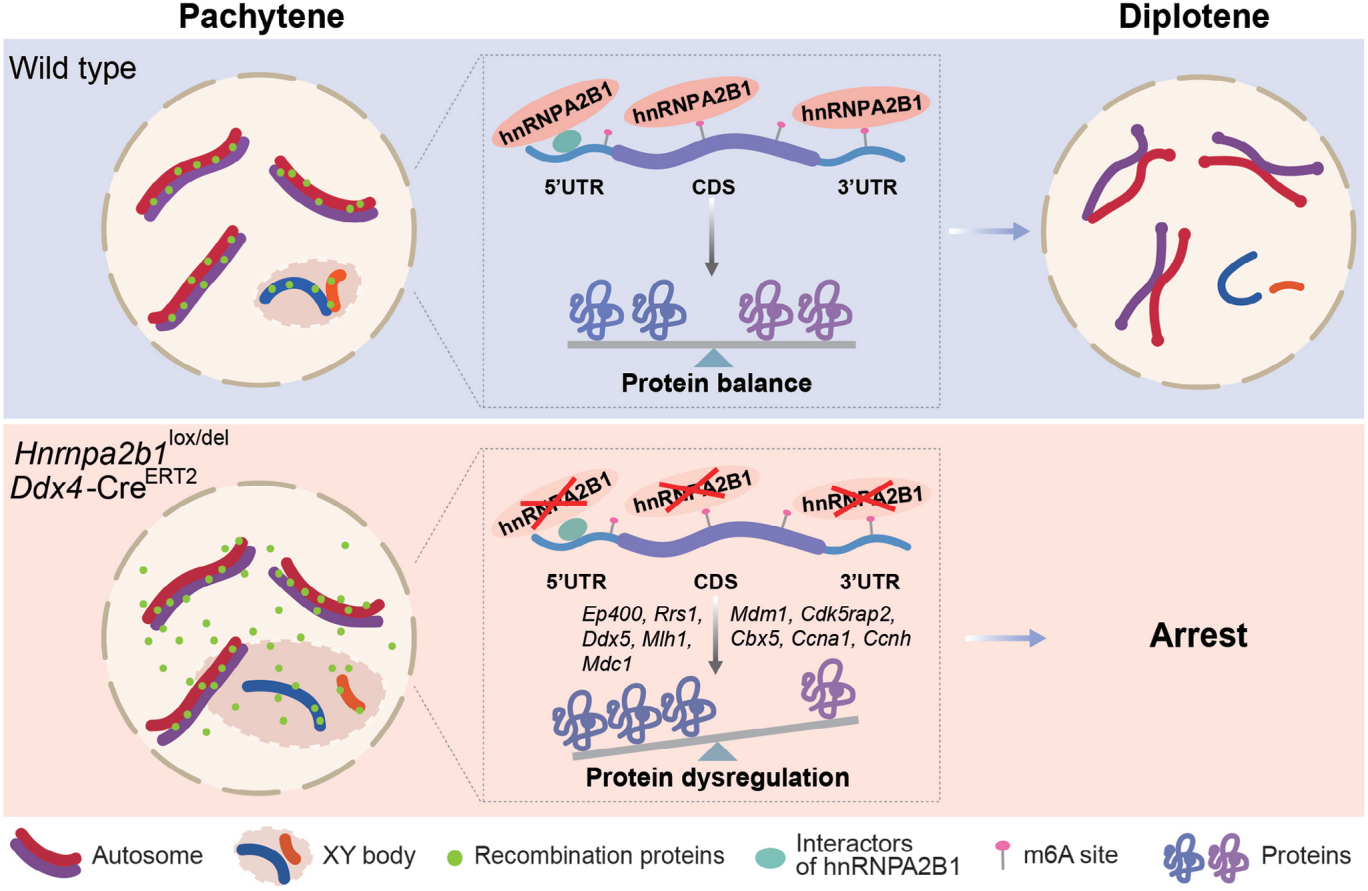
Schematic of pachytene progression regulated by hnRNPA2B1. In the wild type, hnRNPA2B1 interacts with proteins associated with translation and mRNA processing (including eIF4G3, RPS3, RPL13, DDX5, and YTHDC2), and targets some key transcripts related to chromatin organization and remodeling, such as *Ep400* and *Rrs1*, in an m^6^A‐dependent manner, to together ensure progression through late meiotic stages. When hnRNPA2B1 was deleted, the post‐transcriptional process was impaired, with significant protein dysregulation, resulting in meiotic defects, such as expanded sex body, autosome unsynapsis, and recombination foci outside the chromosome axes. Ultimately, pachytene cells were eliminated by the pachytene checkpoint and unable to progress to later stages.

In conclusion, the current study unveils a novel function of the m^6^A reader hnRNPA2B1 in male meiosis. Utilizing a *Ddx4*‐Cre^ERT2^ inducible knockout mouse model, we demonstrated that hnRNPA2B1 interacts with proteins associated with mRNA processing and translation (including eIF4G3, RPS3, RPL13, DDX5, and YTHDC2), and regulates expression of key transcripts involved in meiosis, particularly those related to chromatin organization and remodeling, such as *Ep400* and *Rrs1*, in an m^6^A dependent manner, to ensure progression through late meiotic stages. These findings provide new insights into the regulatory network governing male meiosis and highlight the critical role of hnRNPA2B1 in ensuring its successful completion (**Figure** **8**).

## 5 Experimental Section

### Ethics Statement

All the animal experiments were conducted ethically according to the Guide for the Care and Use of Laboratory Animal guidelines, and were approved by the Institutional Animal Care and Use Committee (animal protocol number: S2795) of Tongji Medical College, Huazhong University of Science and Technology. All mice were housed in specific pathogen‐free (SPF) rooms in the Laboratory of Animal Center, Huazhong University of Science and Technology.

### Mice

All mice were on the C57BL/6J genetic background. *Hnrnpa2b1^flox/+^* mice were purchased from the Cyagen Biosciences company, with the insertion of the Flox site having been made into the interval between exons 2 and 6 of the *Hnrnpa2b1* gene (Cat# S‐CKO11602). *Ddx4*‐Cre^ERT2^ (024760) mouse strains were obtained from The Jackson Laboratory. *Ddx4*‐Cre^ERT2^ is a tamoxifen‐inducible *Cre* that deletes the floxed exons after tamoxifen injection.^[^ ^61^, ^62^ ^]^ As with the previously described mouse breeding strategy, the *Hnrnpa2b1^flox/flox^Ddx4*‐Cre^ERT2^ mice were generated for injection of tamoxifen to obtain inducible *Hnrnpa2b1* knockout mouse models (called *Hnrnpa2b1^iKO^* or iKO). Briefly, for inducible deletion of *Hnrnpa2b1* in germ cells, tamoxifen (Sigma, cat#T5648) was mixed up with corn oil (Sigma, cat#C8267) and injected intraperitoneally daily into 8‐week‐old *Hnrnpa2b1^flox/flox^Ddx4*‐Cre^ERT2^ male mice at a dose of 2mg/30g body weight for five consecutive days or juvenile *Hnrnpa2b1^flox/flox^Ddx4*‐Cre^ERT2^ male mice (P13) for three consecutive days. *Hnrnpa2b1^flox/flox^* male littermates treated with tamoxifen were used as controls. Testes were collected for analysis at different days post‐tamoxifen treatment (dpt). Genotyping of the mice was performed using PCR amplification of genomic DNA extracted from mouse tails. The primers used are listed in Table S4 (Supporting Information).

### Purification of Spermatogenic Cells

STA‐PUT velocity sedimentation was used for purification of pachytene spermatocytes and round spermatids, based on previous study.^[^ ^63^ ^]^ Briefly, to obtain a single testicular cell suspension, mouse testes were excised from control and iKO mice and treated with collagenase IV (BioFroxx, 2091GR001) and trypsin (Beyotime, C0201‐500 mL). The testicular cells were suspended with 0.5% BSA, and was filtered in a linear BSA gradient ranging from 0.5% to 5%. Subsequent to this, different fractions were collected based on cell size, and the purity of pachytene spermatocytes was measured by IF staining co‐staining with SYCP3 and γH2AX. Samples with a purity of more than 80% were used for further analysis, including sequencing, qPCR, and Western blot analysis. For spermatogonia isolation, testes from P5‐P7 mice were digested for testicular single cell suspension, and cultured in a humidified incubator at 37 °C in an atmosphere containing 5% CO_2_. After 2 h incubation, the supernatant cells were collected as spermatogonia.

### Chromatin Fractionation

The nuclear and cytoplasmic proteins were extracted using a Nuclear and Cytoplasmic Protein Extraction Kit (Beyotime Biotechnology, P0027), following the manufacturer's instructions. Briefly, different types of germ cells were dissociated in 200 µl of Buffer A on an ice bath for 15 min. Then, 10 µl of Buffer B was added to the suspension and mixed thoroughly with 5 s of vigorous vortexing followed by an ice bath for 1 min. After centrifugation at 16 000 g for 5 min at 4 °C, the supernatant was transferred to a clean, cold tube to obtain the cytoplasmic proteins. For the nuclear proteins, the sediment was resuspended in 50 µl of nuclear protein extraction reagent containing 1 mM PMSF. This was then vortexed for 15–30 s, followed by an ice bath for 1–2 min, repeated three times. The mixture was then centrifuged at 16 000 g for 10 min at 4 °C, and the supernatant was transferred to a clean cold tube to obtain the nuclear protein.

### Quantitative Real‐Time PCR (qPCR)

RNA from both control and iKO mice was prepared using TRIzol reagent (Invitrogen, 15596‐025) and was reverse‐transcribed using 1st Strand cDNA Synthesis SuperMix for qPCR (Yeasen, 11141ES60), in accordance with the manufacturer's instructions. qPCR was then performed using SYBR green master mix in a Quantagene q225 machine (Kubo Tech, q225‐0370). Statistical analysis was conducted utilizing the 2^‐△△Ct^ method, with normalization performed against *Actb*. All primers used for qPCR are listed in Table S4 (Supporting Information).

### Histology, TUNEL, and Immunofluorescence

Mouse testes and epididymides were fixed in Bouin's solution (Sigma, Lot#SLBJ3855V) at room temperature (RT) for overnight, then embedded in paraffin, and sectioned in 5 µm. The sections were then treated with Periodic acid (BBI, A600690‐0025) and Schiff reagent (Sigma, 1.09033.0500) for stage assignment. For TUNEL and immunofluorescence (IF) assays, testes were fixed in 4% paraformaldehyde, and dehydrated in 5%, 15%, and 30% sucrose solution, and then embedded in O.C.T (Sakura Finetek, 4583). Following antigen retrieval using a citrate solution (pH 6.0), the slides were blocked in 5% normal donkey serum for 1 h at RT. Following this, the slides were incubated with primary and secondary antibodies, after which the slides were mounted with a mounting medium containing DAPI for imaging. PNA (Maokangbio, MP6328) was used to identify tubule stage. Antibodies used in this study are provided in Table S5 (Supporting Information).

### Nuclear Spread Analysis

Spermatocytes were fixed to slides for surface nuclear spread analysis in accordance with the method previously described.^[^ ^64^ ^]^ Briefly, testicular tubules were separated from the testis and placed into hypotonic extraction buffer (HEB buffer, pH 8.2) containing 30 mm Tris, 50 mm sucrose, 17 mm trisodium citrate dihydrate, 5 mm EDTA, 0.5 mm DTT, and 1 mm PMSF for 1.5 h. Then the cells were shaken off repeatedly in 100 ul of 100 mm sucrose buffer (pH 8.2) to make a cell suspension, which was then spread on slides covered with fixation buffer (pH 9.2) containing 1% PFA and 0.15% TritonX‐100. Following an incubation period of over 2 h in a humidity box at RT, the slides were subjected to air‐drying and were then washed twice with 0.4% Photo‐Flo 200 (Kodak). The slides were then stored at −80 °C in preparation for the IF staining. The antibodies used are shown in Table S5 (Supporting Information).

### Immunoprecipitation and Western Blot Analysis

Isolated spermatocytes or GC2 cells were transferred into ice WB/IP Lysis buffer (Beyotime, P0013) and subsequently subjected to centrifugation at 13 000 g for 10 min. Immunoprecipitation was then carried out using Protein A/G Magnetic Beads (MCE, HY‐K0202) with 4 µg of antibody, and this was followed by Mass Spectrometry or Western blot analyses. For IP‐MS analysis, proteins detected in the IP group but not in the IgG group were considered potential interacting proteins. For the Western blot analysis, the protein lysates were loaded on an SDS/PAGE gel and then electroblotted onto a PVDF membrane (Bio‐Rad). The membranes were then incubated with primary and secondary antibodies, and visualization was achieved using the ECL solutions (Bio‐Rad, ClarityTM Western ECL Substrate) and the ChemiDoc XRS+ system (Bio‐Rad). The antibodies used are listed in Table S5 (Supporting Information).

### m^6^A Dot Plot Analysis

The RNA was extracted from the isolated pachytene spermatocytes, and was then denatured at 95 °C for 3 min to disrupt secondary structures. The denatured RNAs were then diluted and crosslinked to Hybond‐N+ membrane (Beyotime, FFN10). The membrane was then washed in TBST for 5 min and blocked in 5% silk milk for 1 h at RT. Following an overnight incubation with an m^6^A antibody at 4 °C, the membrane was washed and incubated with a secondary antibody for 1 h at RT. Visualization was achieved using the ECL solutions (Bio‐Rad, ClarityTM Western ECL Substrate) and a ChemiDoc XRS+ system (Bio‐Rad). The antibodies used are listed in Table S5 (Supporting Information).

### Cell Culture and Transfection, Point Mutation Plasmid Construction, and Luciferase Reporter Assay

The GC2 cells were cultured in DMEM (Procell, PM150210) containing 10% FBS (Albumin Bovine, 4240GR100) and 1% Penicillin‐Streptomycin Solution (Biosharp, BL505A). Cells were cultured in a humidified incubator at 37 °C in an atmosphere containing 5% CO_2_. All siRNA transfection assays were performed using INTERFERin® transfection reagent (Polyplus, 101000036), while overexpression transfection assays were conducted using Lipo8000 transfection reagent (Beyotime, C0533‐1.5 mL).

The CDS/3’UTR regions of the genes of interest were cloned into the expression vector pCMV‐Rluc‐MCS‐Neo (MiaoLingBio, P54803) which contains renilla luciferase. For the mutant reporter plasmid, the adenosine (A) in the m^6^A motif was substituted with guanine (G). For luciferase assay, GC2 cells were seeded into 24‐well plates followed by co‐transfection of wild‐type or mutated reporter plasmids, CMV‐Myc, CMV‐Myc‐hnRNPA2B1‐Mut, CMV‐Myc‐hnRNPA2B1‐WT, and pGL3‐Control plasmids (firefly luciferase reporter vector) (MiaoLingBio, P0195). Following a 46 h period of incubation, the cells were harvested to access the luciferase activity using the Dual‐Lumi^TM^ Luciferase Reporter Gene Assay Kit (Beyotime, RG088S), with the resultant data being normalized to pGL3‐Control. For the siRNA transfection assay, GC2 cells were seeded in 24‐well plates and harvested 72 h after transfection for protein detection, or cultured for 38 h and then used for the overexpression assay. All luciferase activity was measured using a microplate reader (BioTek, 17010919).

### RNA‐Immunoprecipitation (RIP) Sequencing

The RNA immunoprecipitation (RIP) was conducted on isolated pachytene spermatocytes employing the STA‐PUT method. For each sample, 90 ng of total RNA was prepared for RIP‐seq library. The VAHTS Universal V8 RNA seq Library Prep Kit for Illumina (NR605, Vazyme) was used to prepare RIP‐seq chain‐specific libraries through a series of procedures, including fragmentation, first‐strand synthesis, second‐strand synthesis, end repair, linker ligation, amplification, purification, and other steps. Qubit 4.0 (ThermoFisher, Waltham, Massachusetts, USA) was used for library concentration quantification. The RNA‐seq libraries were then subjected to high‐throughput sequencing using the NovaSeq X Plus (Illumina, San Diego, California, USA) in PE150 mode. Following the acquisition of the raw RIP‐seq reads, the quality of these reads was checked using FastQC software (version 0.11.9, https://www.bioinformatics.babraham.ac.uk/projects/fastqc/). Subsequently, the raw paired‐end reads were subjected to trimming and quality control using fastp (version 0.23.4). The quality‐filtered reads were then aligned separately to the reference genome via HISAT2 software (version 2.2.1). The RSeQC (version 5.0.3) was then used for the analysis of reads distribution. The Piranha (version 1.2.1) software^[^ ^65^ ^]^ was used for peak calling, and subsequently, peaks were annotated using the ChIPseeker (version 1.38.0) package.

### RNA‐Sequencing (RNA‐Seq)

The RNA of isolated pachytene spermatocytes was extracted using TRIzol reagent (Invitrogen, 15596‐025) according to the manufacturer's instructions. A total amount of 1.5 µg of total RNA was used for library preparation. The VAHTS mRNA capture beads 2.0 (N403, Vazyme, Nanjing, China) was then utilized for the capture and purification of total RNA. Subsequently, the VAHTS Universal V8 RNA seq Library Prep Kit for Illumina (NR605, Vazyme) was employed for the preparation of RNA‐seq chain‐specific libraries. The quantification of library concentration was performed using Qubit 4.0 (ThermoFisher, Waltham, Massachusetts, USA). The RNA‐seq libraries were then sequenced using NovaSeq X plus (Illumina, San Diego, California, USA) and PE150 mode for high‐throughput sequencing. The quality of the raw reads was checked using the FastQC software (version 0.11.9), and the raw paired‐end reads were subjected to trimming and quality control using the fastp (version 0.23.4) with default parameters. The quality‐filtered reads were then aligned separately to the reference genome via HISAT2 software (version 2.2.1). Transcriptome changes were analyzed by DESeq2 package (version 1.42.1) and curated by both manual and automated methods. Differentially expressed genes (DEGs) were identified with the criteria of a |Log_2_ (Fold Change)| ≥ 0.5 and *p*‐value < 0.05.

### Proteomic Analysis

Isolated pachytene spermatocytes were subjected to a lysis buffer (1% SDC/100 mm Tris‐HCl, pH 8.5). Following ultrasonication, the mixture was centrifuged at 12 000 g for 5 min, and protein concentration of the supernatant was then determined by BCA method. With equivalent protein concentrations maintained across different samples, the lysis buffer was added to achieve a uniform volume. Protein reduction and alkylation were conducted with TCEP and CAA at 37 °C for 1 h. Urea was diluted below 2 m using 100 mm Tris‐HCl. Subsequently, trypsin was added at a ratio of 1:50 (enzyme:protein, w/w) for overnight digestion at 37 °C. Then, the pH was adjusted to 6.0 using TFA. Following centrifugation, the supernatant was subjected to peptide purification using a self‐made SDB‐RPS desalting column. Subsequent analysis of all samples was conducted on a timsTOF Pro (Bruker Daltonics), a hybrid trapped ion mobility spectrometer (TIMS) quadrupole time‐of‐flight mass spectrometer. An UltiMate 3000 RSLCnano system (Thermo) was coupled to timsTOF Pro with a CaptiveSpray nano ion source (Bruker Daltonics). Peptide samples were injected into a C18 Trap column (3 µm particle size, 100 Å pore size, Thermo), and separated in a reversed‐phase C18 analytical column (75 µm × 15 cm, 1.7 µm particle size, 100 Å pore size, IonOpticks). The separation gradient was established using Mobile phase A (0.1% formic acid in water) and Mobile phase B (0.1% formic acid in ACN), with a flow rate of 300 nL min^−1^. The MS were operated in diaPASEF mode, with the capillary voltage set at 1500 V. The MS and MS/MS spectra were acquired from 100 to 1700 m z^−1^. With default parameters set in LS/MS, DIA raw data were analyzed with DIA‐NN (DIA‐NN 1.8.1) in library‐free mode. Spectra files were then searched against the Mouse protein sequence database (2024‐09‐10, 17217 entries) from Uniprot. The bioinformatics analysis was conducted in RStudio.

### Statistical Analysis

All quantitative data are presented as mean ± SD. Significance was tested using the two‐tailed unpaired Student's *t*‐test (*p < 0.05, ** p < 0.01, *** p < 0.001, and **** p < 0.0001) with Prism 9.0 (GraphPad Software). Data analysis was carried out using RStudio 2023.06.1 + 524, or Metascape.^[^ ^66^ ^]^ Image processing was done using Photoshop (Adobe) and ImageJ (NIH).

## Conflict of Interest

The authors declare no conflict of interest.

## Author Contributions

L.Y., Y.Z., B.Z., J.Z., and M.X. contributed equally to this work. L.Y. and S.Y. conceived and designed the study. L.Y., Y.Z., B.Z., J.Z., M.X., N.J., J.X., H.G., W.X., X.W., and F.W. performed most bench work and data analyses. L.Y. wrote the manuscript. S.Y. supervised the project and revised the manuscript. All authors read and approved the manuscript.

## Supporting information



Supporting Information

Supplemental Table 1

Supplemental Table 2

Supplemental Table 3

Supplemental Table 4

Supplemental Table 5

## Data Availability

The data that support the findings of this study are available from the corresponding author upon reasonable request.
