# Supplementary material for: m6A Reader hnRNPA2B1 Modulates Late Pachytene Progression in Male Meiosis Through Post‐Transcriptional Control
Source: Adv Sci (Weinh). 2025 Jul 28;12(38):e06600. doi: 10.1002/advs.202506600 (PMC12520546; doi:10.1002/advs.202506600)
Supplement: Supplementary file 1 — Supporting Information [file ADVS-12-e06600-s004.pdf]

ADVANCED SCIENCE
Open Access

Supporting Information

for *Adv. Sci.*, DOI 10.1002/advs.202506600

m^{6}A Reader hnRNPA2B1 Modulates Late Pachytene Progression in Male Meiosis Through Post-Transcriptional Control

*Lisha Yin, Yuting Zhang, Bingqian Zhang, Jin Zhang, Mengneng Xiong, Nan Jiang, Jinxin Xiao, Huihui Gao, Wenjing Xiong, Xiaoli Wang, Fengli Wang and Shuiqiao Yuan**

Supporting Information

m^6A reader hnRNPA2B1 modulates late pachytene progression in male meiosis through post-transcriptional control

Lisha Yin, et. al.

Figure S1 (Related to Figure 1)

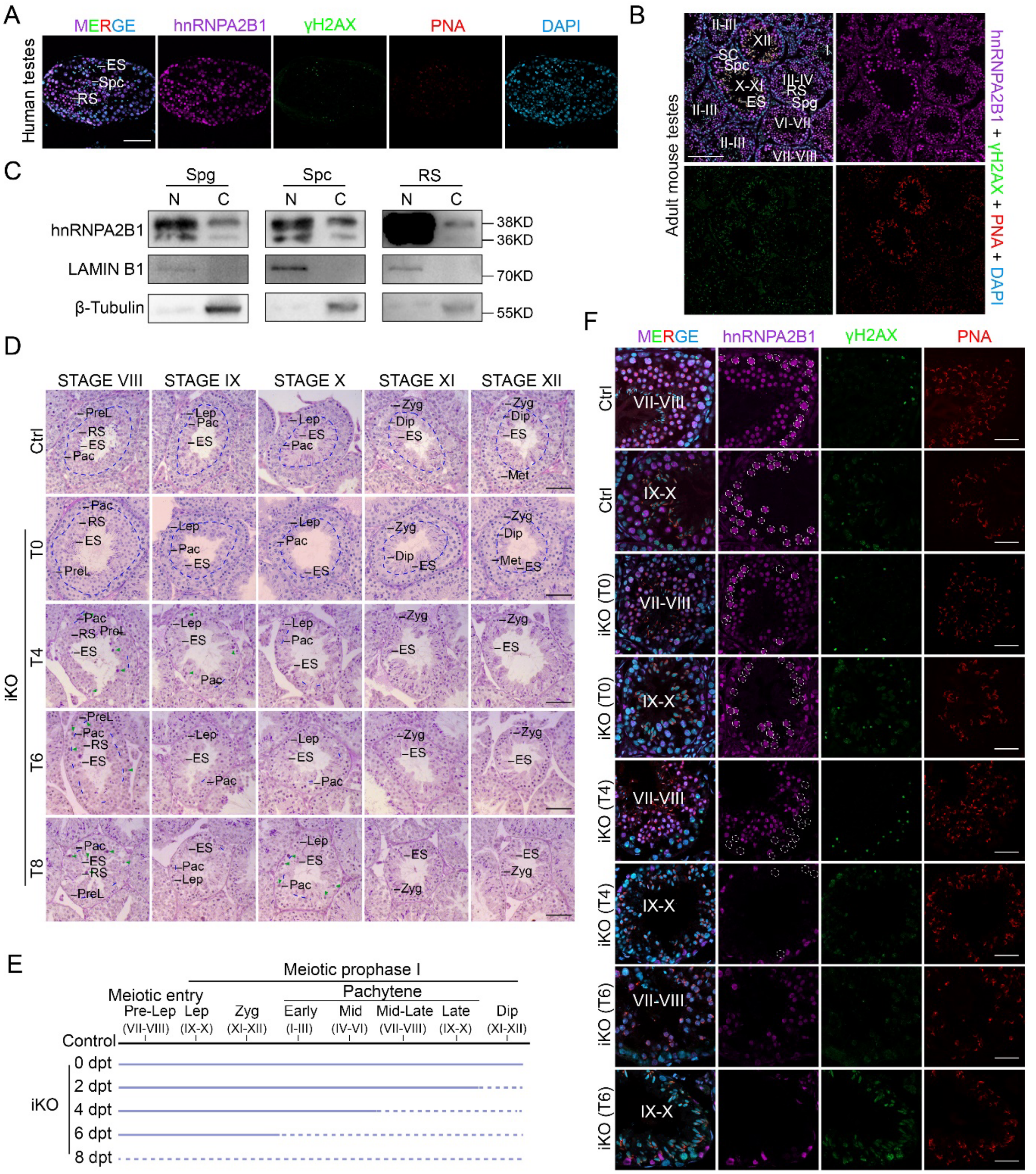

Figure S1. hnRNPA2B1 is essential for late meiotic progression, especially pachytene stage.

(**A**) hnRNPA2B1 expression pattern in human testis. γH2AX was used for spermatocytes identification, while PNA was used to identify round or elongating spermatids. Scale bars = 50 μm.

(**B**) hnRNPA2B1 expression pattern in mouse testis. γH2AX was used for spermatocytes identification, while PNA was used for staging tubules. Scale bars = 100 μm.

(**C**) Western blot assay of protein expression pattern of hnRNPA2B1 in nucleus (N) and cytoplasm (C) of spermatogonia (Spg), spermatocytes (Spc), and round spermatids (RS), respectively. For western blot, LAMIN B1 and β-Tubulin serve as the loading controls.
(**D**) Histological analyses of different stages of testicular sections from Ctrl and *Hnrnpa2b1*^{iKO} (iKO) adult mice at 0 dpt (T0), 4 dpt (T4), 6 dpt (T6), 8 dpt (T8) are shown. Seminiferous tubules at stage VIII-XII are shown, and the dashed blue lines indicate the layer of pachytene/diplotene spermatocytes. Scale bars = 50 μm.
(**E**) Schematics of meiotic cell loss in prophase I stage in *Hnrnpa2b1*^{iKO} mice at T0, T2, T4, T6, and T8. The dashed lines indicate obvious cell loss.
(**F**) Immunofluorescence to confirm the knockout efficiency of hnRNPA2B1 in pachytene spermatocytes of *Hnrnpa2b1*^{iKO} mice at T0, T4 and T6. PNA was used to identify tubule stage. Representative images of tubules at Stage VII-VIII and Stage IX-X are shown. White lines indicate pachytene spermatocytes. Scale bars = 50 μm.

Figure S2 (Related to Figure 2)

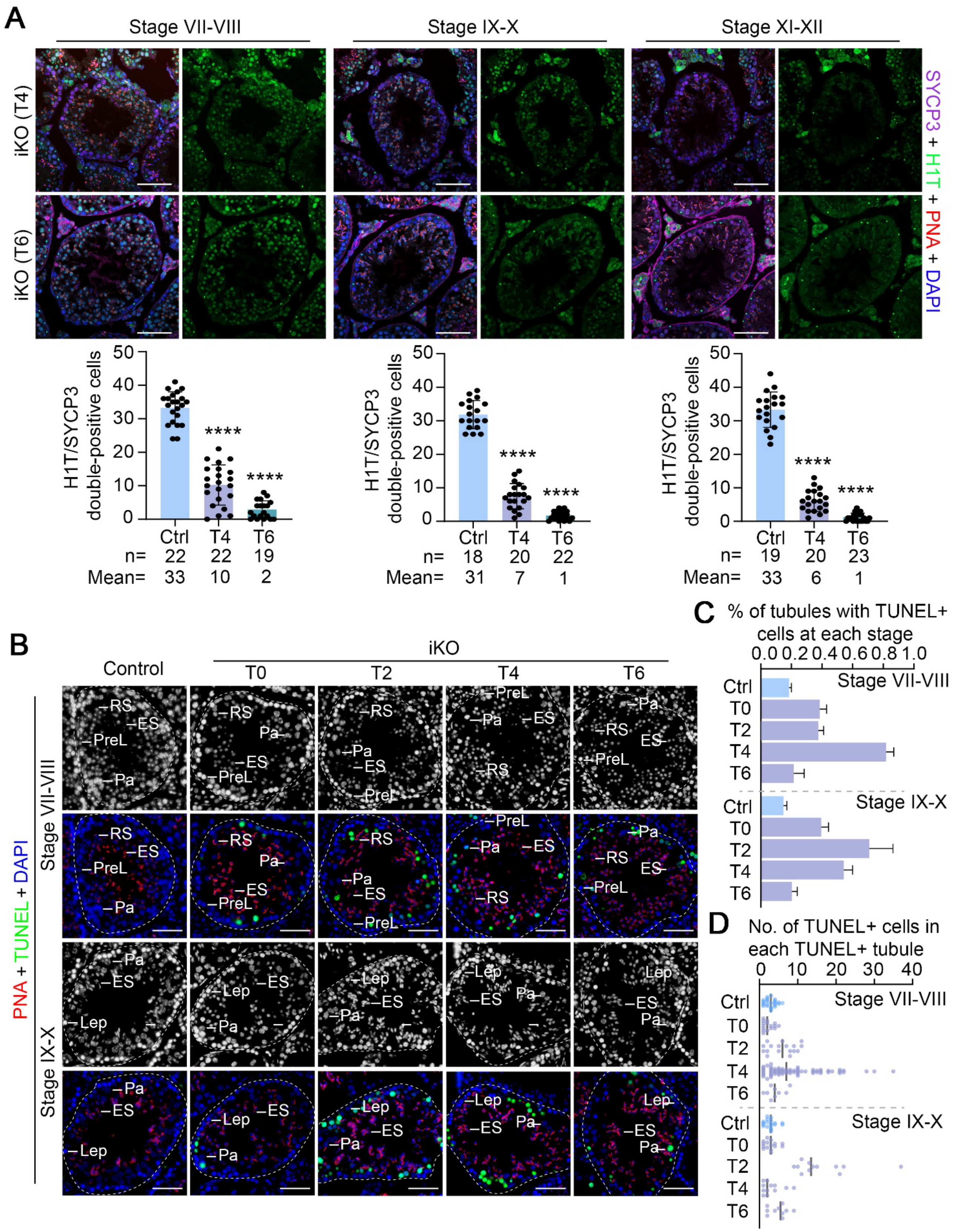

Figure S2. Loss of hnRNPA2B1 leads to late pachytene spermatocyte apoptosis.
(**A**) Immunofluorescence and quantification of SYCP3/H1T for pachytene or diplotene cell

number detection in *Hnrnpa2b1*^{iKO} mice at T4 and T6. PNA was used to identify tubule stage. n, the total number of seminiferous tubules from Ctrl and *Hnrnpa2b1*^{iKO} testes. Mean, the average number of H1T/SYCP3 double-positive cells in seminiferous tubules. Three males per genotype (Ctrl and iKO) were analyzed. Scale bars = 50 μm. The quantified data are presented as mean ± SD. ****P < 0.0001.

(**B**) TUNEL analysis of testis sections from Ctrl and *Hnrnpa2b1*^{iKO} mice at T0, T2, T4, and T6. The white dashed lines demarcate the boundary of seminiferous tubules. PNA was used to identify tubule stage. Scale bars = 50 μm.

(**C**) Percentage of TUNEL-positive tubules from Ctrl and *Hnrnpa2b1*^{iKO} testes at T0, T2, T4, and T6. Two males per genotype (Ctrl and iKO) were analyzed.

(**D**) Quantification of TUNEL-positive cells in TUNEL-positive tubules from Ctrl and *Hnrnpa2b1*^{iKO} testes at T0, T2, T4, and T6. Two males per genotype (Ctrl and iKO) were analyzed.

Figure S3 (Related to Figure 3-4)

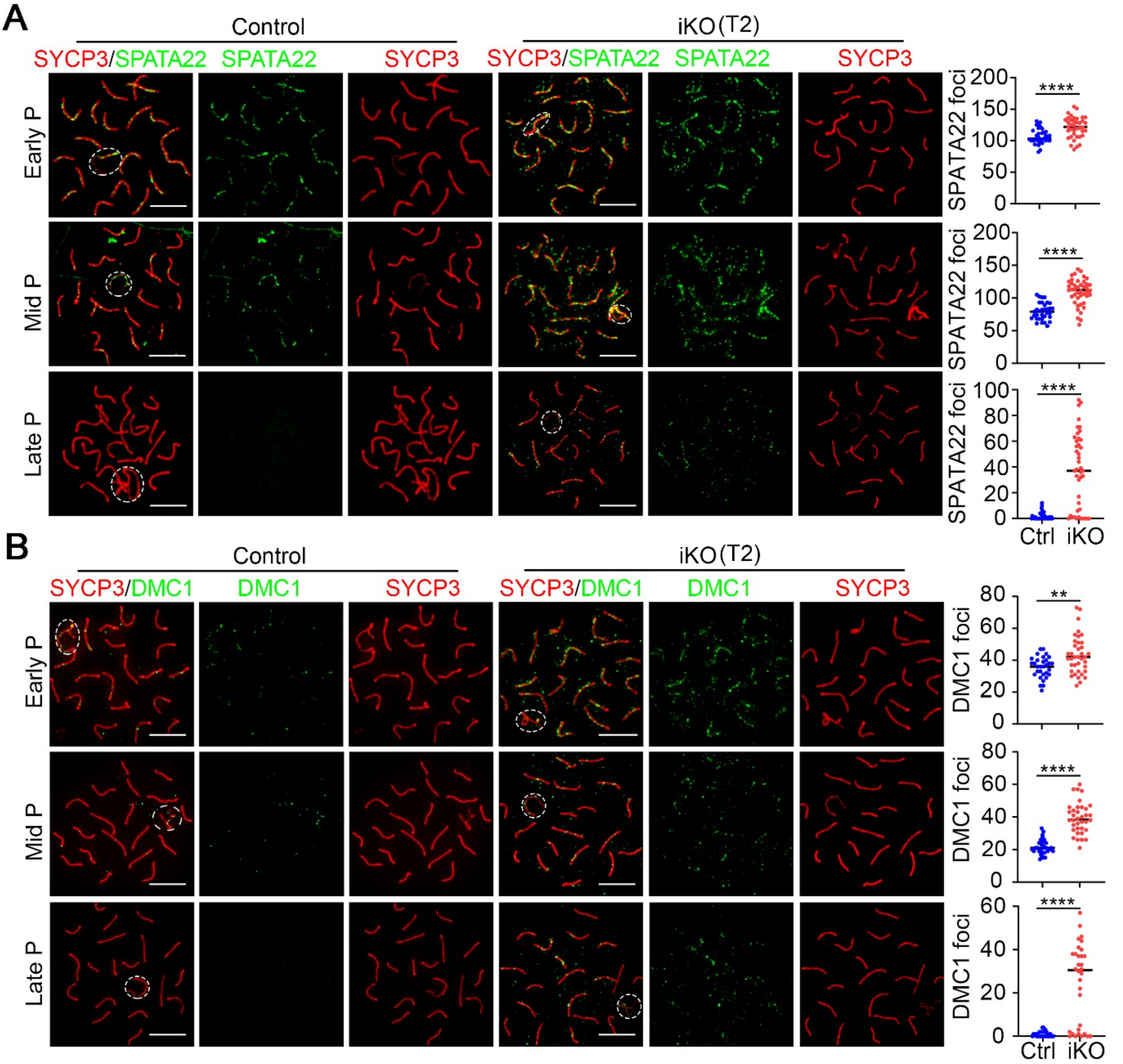

Figure S3. Off-axis foci of recombination repair related proteins in hnRNPA2B1-depleted pachytene spermatocytes.

(**A-B**) Chromosome spread analysis and quantification of SPATA22 (A) and DMC1 (B) in Ctrl and *Hnrnpa2b1*^{iKO} pachytene spermatocytes at T2. The white dashed lines indicate sex body. Abbreviations: Early P, early pachytene; Mid P, mid pachytene; Late P, late pachytene. Scale bars = 10 μm. Three males per genotype (Ctrl and iKO) were analyzed. The quantified data are presented as mean ± SD. $^{**}P < 0.01$, $^{****}P < 0.0001$.

Figure S4 (Related to Figure 3-4)

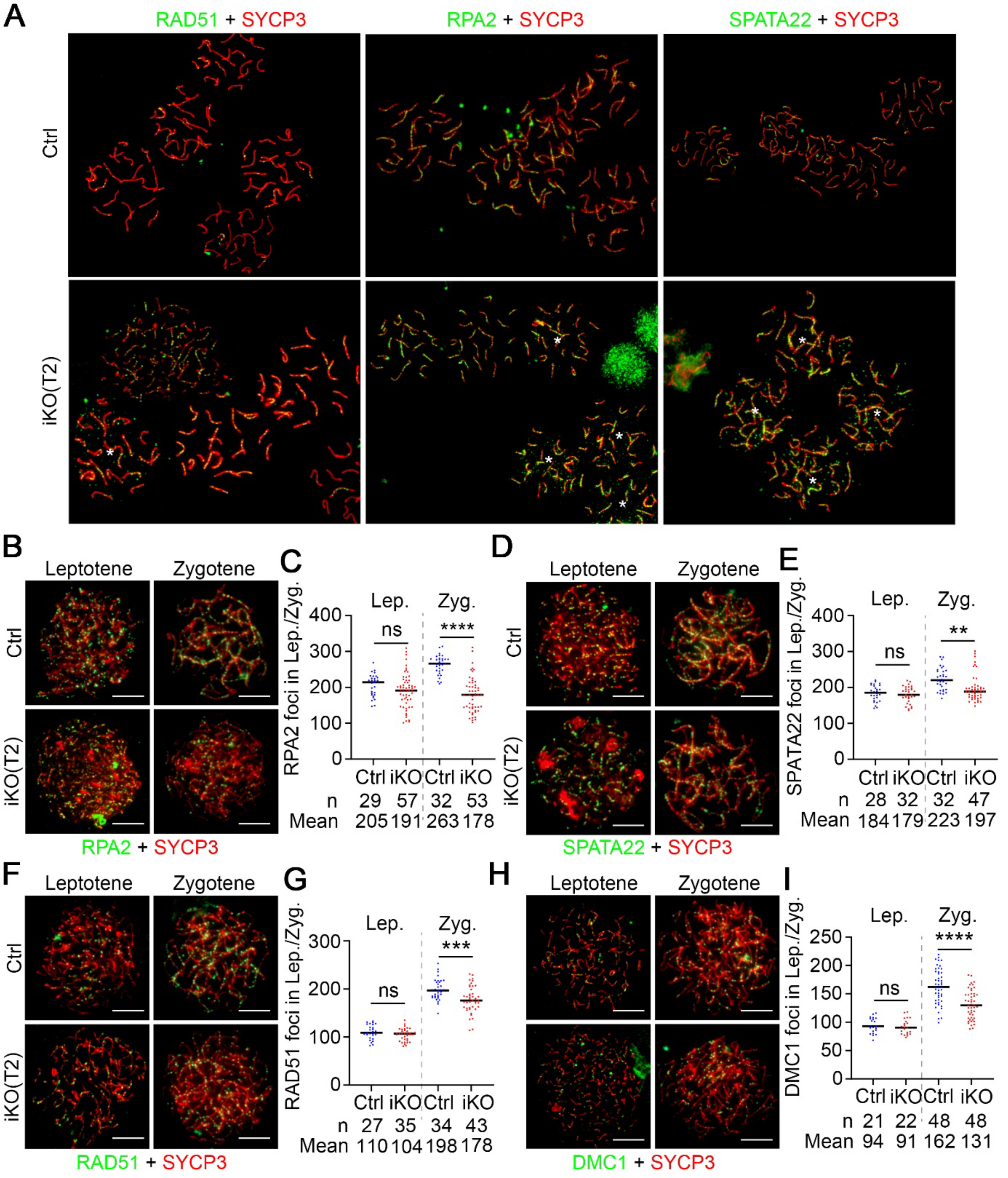

Figure S4. Recruitment of recombination related proteins to chromosome axes in early meiotic spermatocytes was unaffected by hnRNPA2B1 deletion.

(**A**) Wide-field image of immunostaining with RPA2, SPATA22, and RAD51 on chromosome spread slides from control and *Hnrnpa2b1*^{iKO} mice at T2. White asterisks meant spermatocytes with off-axis signals.

(**B-I**) Chromosome spread assays (B, D, F, H) and quantification (C, E, G, I) of homologous

repair proteins RPA2, SPATA22, RAD51, and DMC1 in early meiotic spermatocytes (leptotene and zygotene spermatocytes) of Ctrl and *Hnrnpa2b1*^{iKO} mice at T2. Scale bars = 10 μm. The quantified data are presented as mean ± SD. **P < 0.01, ***P < 0.001, ****P < 0.0001. ns, not significant. n, the total number of quantified pachytene spermatocytes. Mean, the average number of RPA2, SPATA22, RAD51, and DMC1 foci in Ctrl and *Hnrnpa2b1*^{iKO} mice at T2. Three males per genotype (Ctrl and iKO) were analyzed.

Figure S5 (Related to Figure 3-4)

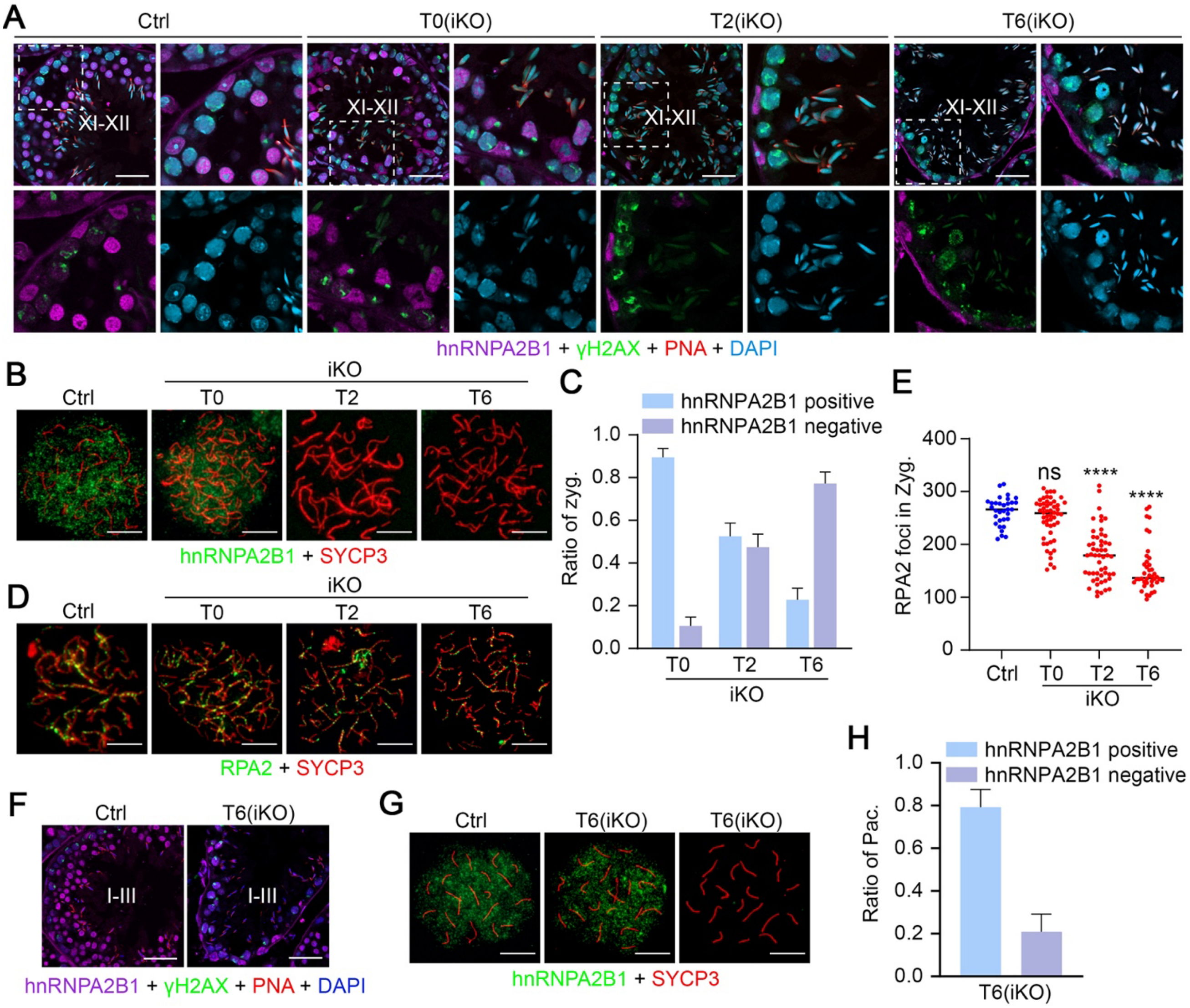

Figure S5. Early meiotic cells with hnRNPA2B1 deletion leads to zygotene arrest.

(**A**) Immunofluorescence of hnRNPA2B1, γH2AX, and PNA to assess the knockout efficiency of zygotene cells in *Hnrnpa2b1*^{iKO} mice at T0, T2 and T6. PNA was used to identify tubule stage. The white dashed boxes meant the enlarged region. Scale bars = 50 μm.

(**B-C**) Chromosome spread assays (B) and quantification (C) of hnRNPA2B1 in zygotene cells of control and *Hnrnpa2b1*^{iKO} mice at T0, T2 and T6. Scale bars = 10 μm. Three males per group were analyzed.

(**D-E**) Chromosome spread assays (D) and quantification (E) of RPA2 in zygotene cells of control and *Hnrnpa2b1*^{iKO} mice at T0, T2 and T6. Scale bars = 10 μm. The quantified data are presented as mean ± SD. ****$P < 0.0001$. ns, not significant. Three males per group were analyzed.

(**F**) Immunofluorescence of hnRNPA2B1, γH2AX, and PNA to assess the knockout efficiency of early pachytene cells in *Hnrnpa2b1*^{iKO} mice at T6. PNA was used to identify tubule stage. Scale bars = 50 μm.

(**G-H**) Chromosome spread assays (G) and quantification (H) of hnRNPA2B1 in pachytene

cells of control and *Hnrnpa2b1*^{iKO} mice at T6. Scale bars = 10 μm. Three males were analyzed.

Figure S6 (Related to Figure 3-4)

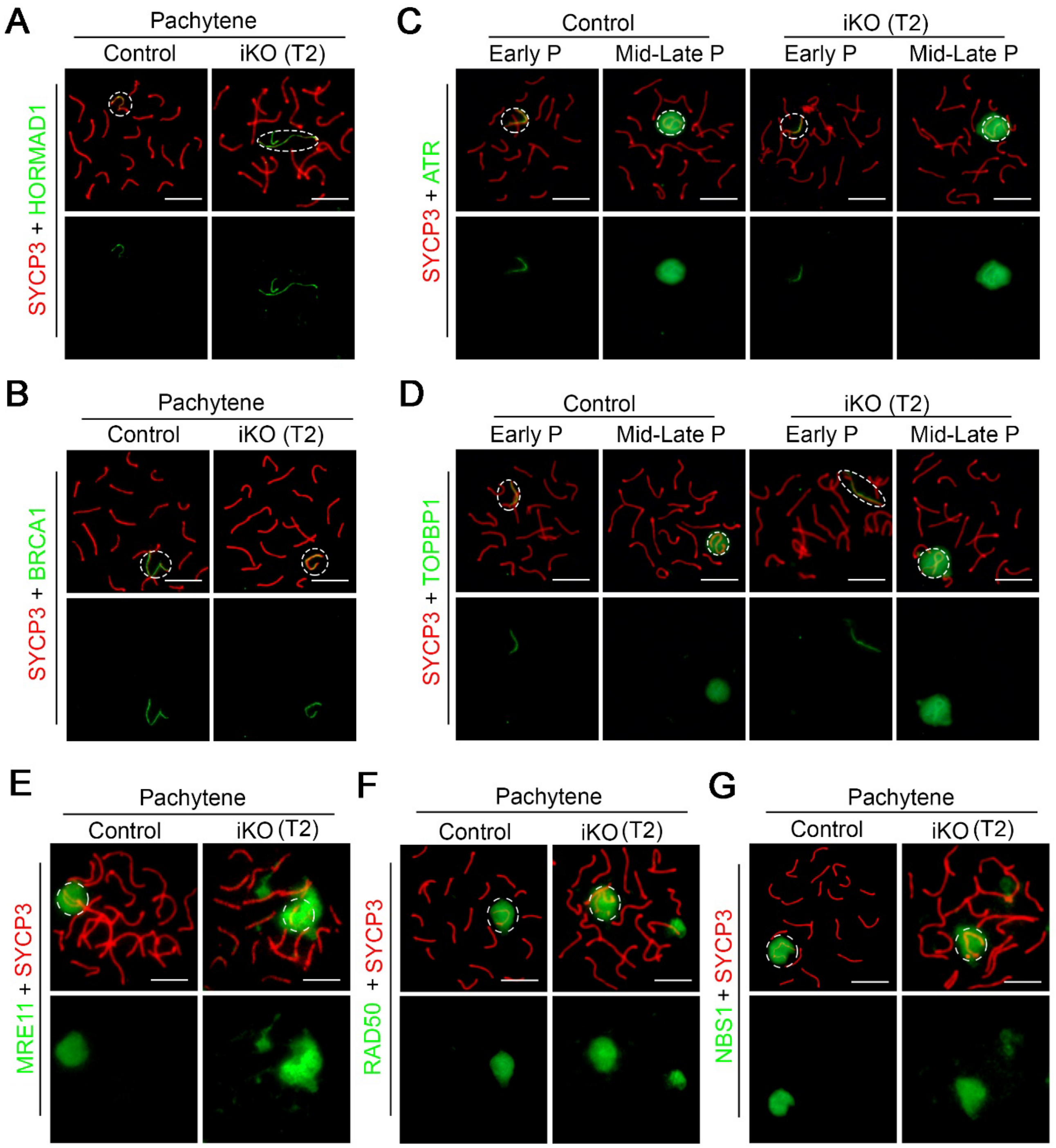

Figure S6. Localization of recombination surveillance proteins.
(**A-G**) Chromosome spread analysis of DNA damage response (DDR) factors HORMAD1 (A), BRCA1 (B), ATR (C), and TOPBP1 (D), and DSB sensors MRE11 (E), RAD50 (F), and NBS1 (G) in Ctrl and *Hnrnpa2b1*^{iKO} pachytene spermatocytes at T2. The white dashed lines indicate sex body. Abbreviations: Early P, early pachytene; Mid-Late P, mid to late pachytene. Scale bars = 10 μm.

Figure S7 (Related to Figure 1-4)

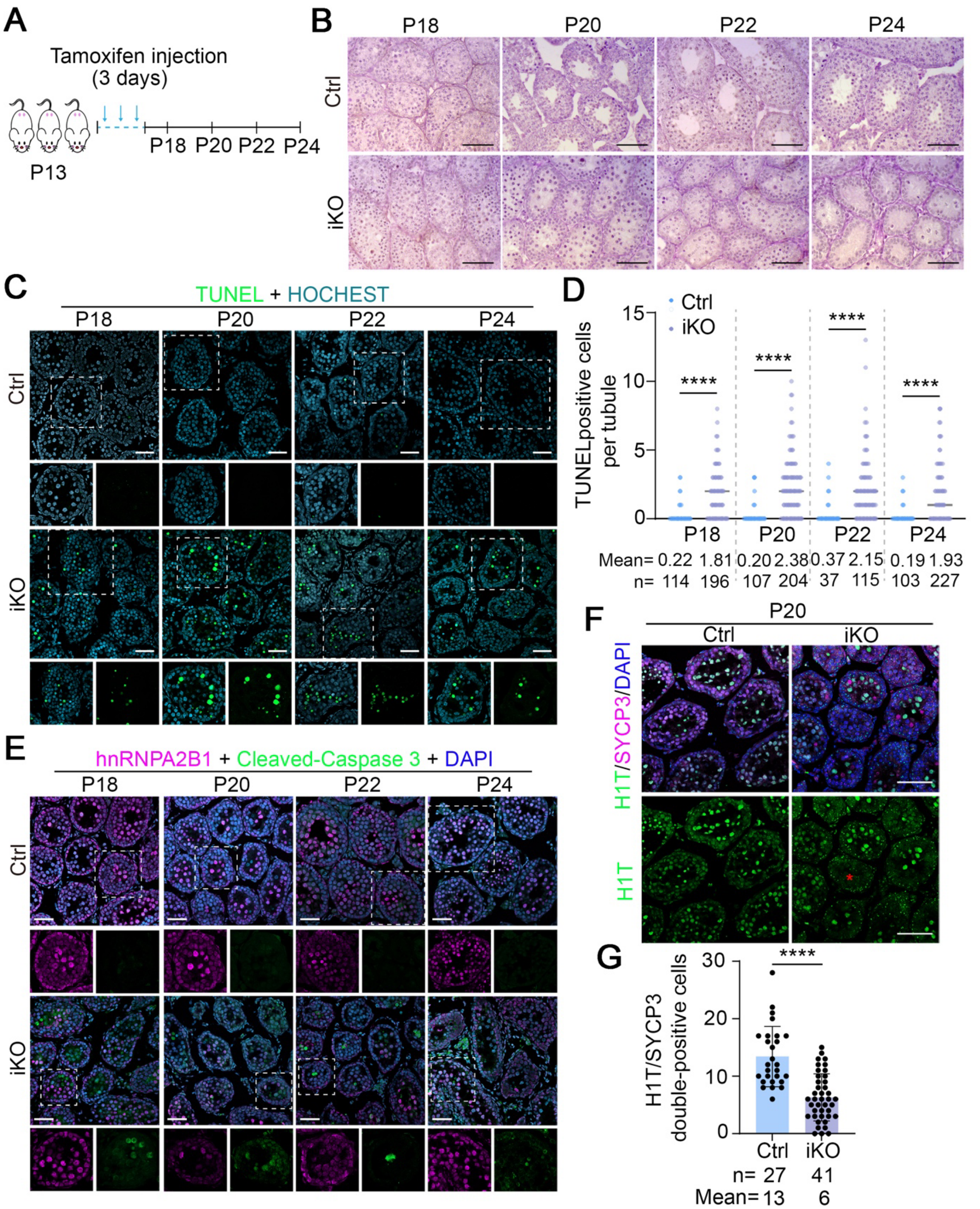

Figure S7. hnRNPA2B1 is essential for pachytene progression in the first wave of spermatogenesis.

(**A**) Schematics of *Hnrnpa2b1*$^{flox/flox}$ *Ddx4*-Cre^{ERT2} juvenile iKO mice generation. Ctrl (*Hnrnpa2b1*$^{flox/flox}$) and *Hnrnpa2b1*$^{flox/flox}$ *Ddx4*-Cre^{ERT2} juvenile mice at P13 were treated with

three consecutive days tamoxifen injection, and the testis tissues were collected at P18, P20, P22, and P24 for investigation.

(**B**) Histological analysis of seminiferous tubules at P18, P20, P22, and P24 Ctrl and iKO juvenile mice. Scale bars = 100 μm.

(**C-D**) TUNEL assay (C) and quantification (D) for apoptosis confirmation in Ctrl and iKO juvenile mice at P18, P20, P22, and P24. The white dashed lines indicate representative tubules. Scale bars = 50 μm. The quantified data are presented as mean ± SD. ****$P < 0.0001$. n, the total number of quantified seminiferous tubules. Mean, the average number of TUNEL positive cells per tubule in Ctrl and *Hnrnpa2b1*^{iKO} mice. Three males per genotype (Ctrl and iKO) were analyzed.

(**E**) Immunofluorescence of hnRNPA2B1 and Cleaved-Caspase 3 for apoptosis confirmation in Ctrl and iKO juvenile mice at P18, P20, P22, and P24. The white dashed lines indicate representative tubules. Scale bars = 50 μm.

(**F-G**) Immunofluorescence (F) and quantification (G) of SYCP3/H1T for pachytene or diplotene cell number detection in Ctrl and iKO juvenile mice at P20. Scale bars = 50 μm. Red asterisk, tubules without H1T positive cells. The quantified data are presented as mean ± SD. ****$P < 0.0001$. n, the total number of seminiferous tubules from Ctrl and *Hnrnpa2b1*^{iKO} testes. Mean, the average number of H1T/SYCP3 double-positive cells in seminiferous tubules. Three males per genotype (Ctrl and iKO) were analyzed.

Figure S8 (Related to Figure 5-6)

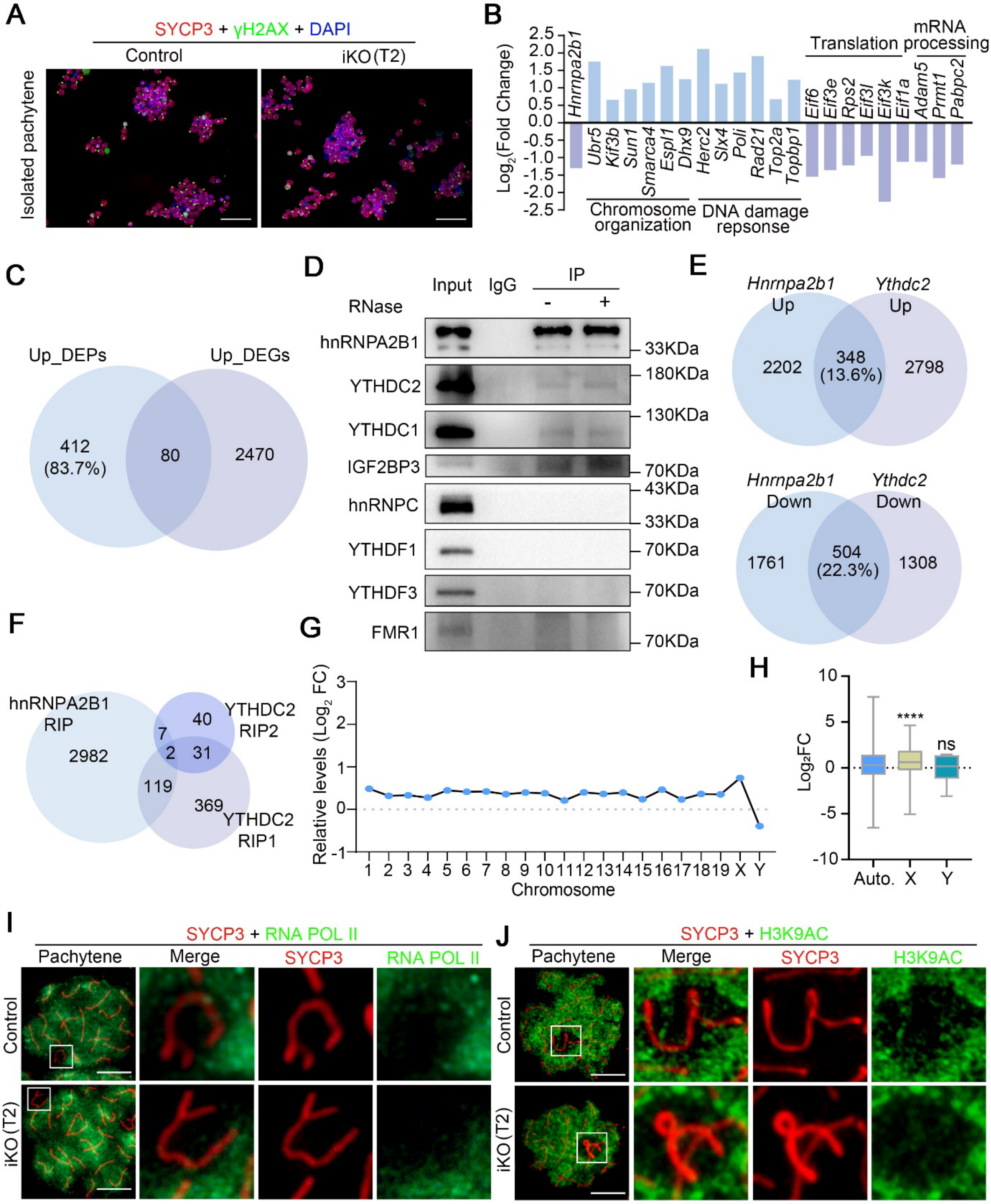

Figure S8. Interactions between hnRNPA2B1 and m⁶A readers, and normal MSCI in hnRNPA2B1-depleted pachytene spermatocytes are shown.

(**A**) Purity confirmation of pachytene spermatocytes isolated from STA-PUT method. Scale bars = 100 μm.

(**B**) Analysis of Log_2(Fold change) of some selected genes identified by RNA-seq.
(**C**) Overlap of up-regulated DEGs and up-regulated DEPs.
(**D**) Co-IP to assess the interactions between hnRNPA2B1 and other m^6A readers.
(**E**) Comparisons between DEGs in *Hnrnpa2b1*^{iKO} pachytene spermatocytes and *Ythdc2*^{iKO} pachytene spermatocytes.
(**F**) Overlaps of hnRNPA2B1-targeted genes and published YTHDC2-targeted genes. YTHDC2-RIP1 and YTHDC2-RIP2 data were from [1] and [2], respectively.
(**G**) Average transcriptome changes on each chromosome based on RNA-seq data. For each chromosome, the average expression levels were calculated by summing the Log_2FC values of all detectable genes.
(**H**) Analysis of transcript levels in autosomes, X, and Y, based on RNA-seq data. The quantified data are presented as mean ± SD. ****$P < 0.0001$. ns, not significant.
(**I-J**) Chromosome spread assay of RNA POL II (I) and H3K9AC (J) in Ctrl and *Hnrnpa2b1*^{iKO} pachytene spermatocytes. The white lines demarcated sex chromosomes. Scale bars = 10 μm.

Figure S9 (Related to Figure 5)

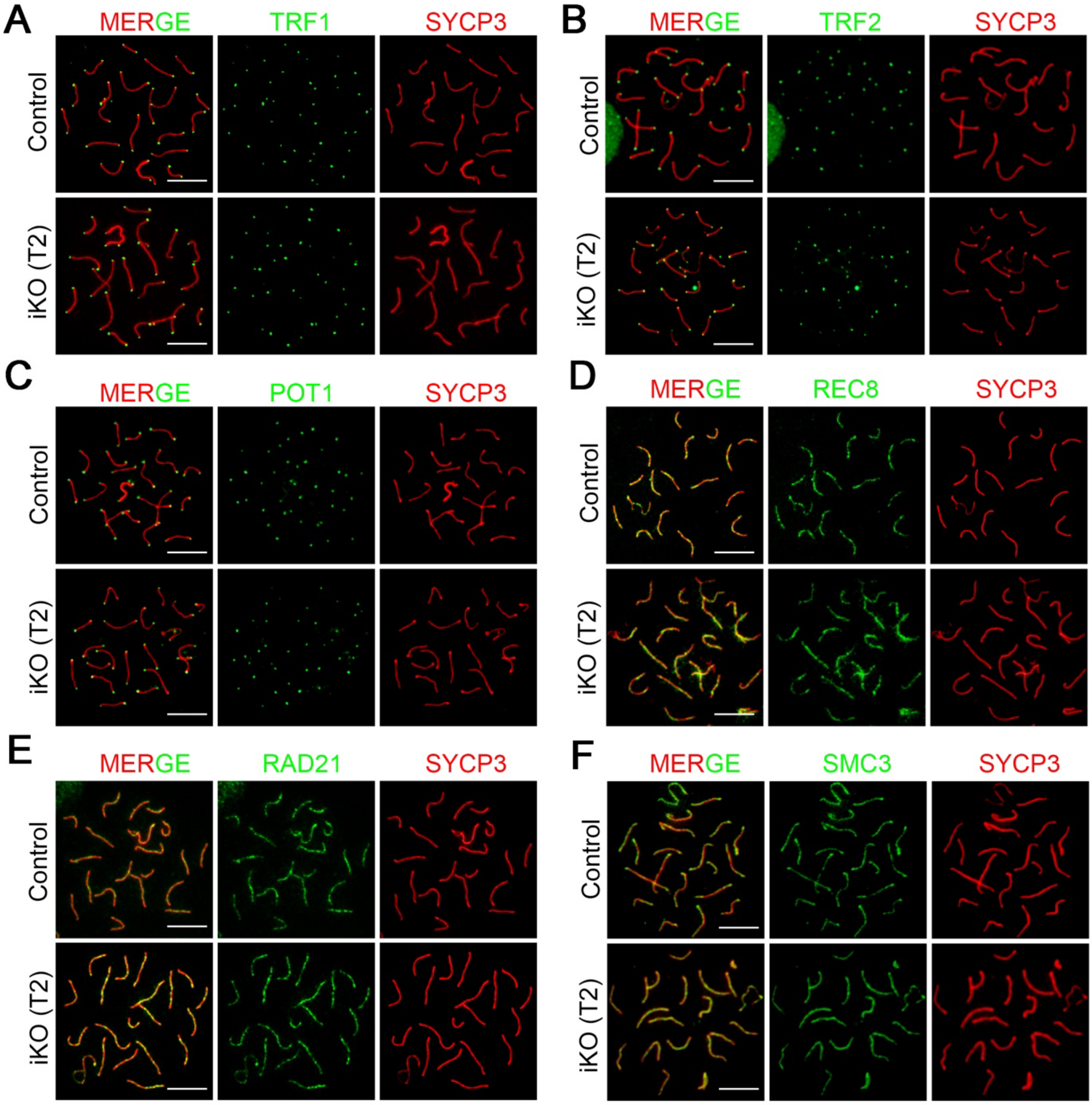

Figure S9. Normal telomere structure and behavior, and cohesin subunits localization in hnRNPA2B1-depleted pachytene spermatocytes are shown.

(**A-C**) Chromosome spread analysis of telomeric proteins TRF1 (A), TRF2 (B), and POT1 (C) in Ctrl and *Hnrnpa2b1*^{iKO} pachytene spermatocytes at T2. Scale bars = 50 μm.

(D-F) Chromosome spread analysis of cohesin subunits REC8 (D), RAD21 (E), and SMC3 (F) in Ctrl and *Hnrnpa2b1*^{iKO} pachytene spermatocytes at T2. Scale bars = 50 μm.

References:

[1] P.J. Hsu, Y. Zhu, H. Ma, Y. Guo, X. Shi, Y. Liu, M. Qi, Z. Lu, H. Shi, J. Wang, Y. Cheng, G. Luo, Q. Dai, M. Liu, X. Guo, J. Sha, B. Shen, C. He, Ythdc2 is an N(6)-methyladenosine binding protein that regulates mammalian spermatogenesis, Cell Res, 27 (2017) 1115-1127.
[2] Y.Q.S. Soh, M.M. Mikedis, M. Kojima, A.K. Godfrey, D.G. de Rooij, D.C. Page, Meioc maintains an extended meiotic prophase I in mice, PLoS Genet, 13 (2017) e1006704.
